# Magnetic Silica Nanosystems With NIR-Responsive and Redox Reaction Capacity for Drug Delivery and Tumor Therapy

**DOI:** 10.3389/fchem.2020.567652

**Published:** 2020-10-22

**Authors:** Chengzheng Jia, Hang Wu, Keyi Luo, Weiju Hao, Shige Wang, Mingxian Huang

**Affiliations:** ^1^College of Science, University of Shanghai for Science and Technology, Shanghai, China; ^2^Department of General Surgery, Xinhua Hospital, Shanghai Jiaotong University School of Medicine, Shanghai, China

**Keywords:** photothermal therapy, Fenton reaction, chemodynamic therapy, magnetic nanoparticles, drug delivery

## Abstract

In recent years, more and more researches have focused on tumor photothermal therapy and chemodynamic therapy. In this study, we prepared a multifunctional nanomaterial with potential applications in the above area. The Fe_3_O_4_ nanoparticles were synthesized with suitable size and uniformity and then coated with mesoporous silica and polydopamine. The unique core-shell structure not only improves the drug loading of the magnetic nanomaterials, but also produces high photothermal conversion efficiency. Furthermore, the reducibility of polydopamine was found to be able to reduce Fe^3+^ to Fe^2+^ and thus promote the production of hydroxyl radicals that can kill the tumor cells based on the Fenton reaction. The magnetic nanomaterials are capable of simultaneously combining photothermal and chemodynamic therapy and permit the efficient treatment for tumors in the future.

## Introduction

Cancer is one of the leading causes of death in the world, especially malignant tumors, which poses a great threat to the safety of human life (Siegel et al., 2016). As a delivery system for therapeutic drugs, nanocarriers show a huge potential in cancer treatment. With the continuous development of nanotechnology, the genetic and drug loading capabilities of nanomaterials have received widespread attention (Jia et al., 2015). It has been a continuous effort to explore the novel preparation methods of nanomaterials for the effective therapy of tumors in the future (Qin et al., 2017). However, up to now, only a limited number of nanocarriers have been successfully used in the clinical treatment of cancer (Bulbake et al., 2017), because many nanocarriers either have a low drug-carrying capacity or have difficulty to efficiently reach the tumor site (Jia et al., 2015). The ideal nanocarrier should have a high drug loading efficiency and a suitable size, which can accurately transport the drug to the target area in the body for controlled release (Liu et al., 2014; Bose et al., 2016; Li et al., 2018).

In recent years, more and more functional materials have been introduced into the nanotechnology, and many new treatment methods have emerged, such as photothermal therapy (PTT), magnetocaloric therapy, and chemodynamic therapy (CDT) (Du et al., 2017; Chen et al., 2019; Wu et al., 2019; Zhou et al., 2020). These new treatments have greatly expanded the application of nanocarriers in the treatment of tumors. PTT is a new type of therapy with low toxicity, high efficiency, and safety (Huang et al., 2017; Gulzar et al., 2018). Tumor tissue has enhanced permeability and retention than the normal one. Many nanomaterials with an appropriate size and photothermal conversion ability are used for tumor treatment (Huo et al., 2017; Wang et al., 2019, 2020). During PTT, tumor cells are killed by the heat of photothermal nanoparticles after being irradiated with near-infrared (NIR) laser (Li et al., 2018; Lin et al., 2018; Tiwari et al., 2019). PTT involves the use of photothermal materials that can effectively convert light radiation into heat to cure cancer via hyperthermia (Feng et al., 2018a). In the past few years, many documents have reported various materials with photothermal effects, such as metal sulfide, MnO_2_, carbon-based graphene, precious metal Au, and the organic polypyrrole, polyaniline, polydopamine (PDA), etc (Jahanban-Esfahlan et al., 2015; Wang et al., 2015; Song et al., 2017; Feng et al., 2018b; Xu et al., 2018, 2020). However, many of them have drawbacks, and satisfactory therapeutic effects cannot be obtained (Feng et al., 2018b; Xue et al., 2019).

**Scheme 1:**
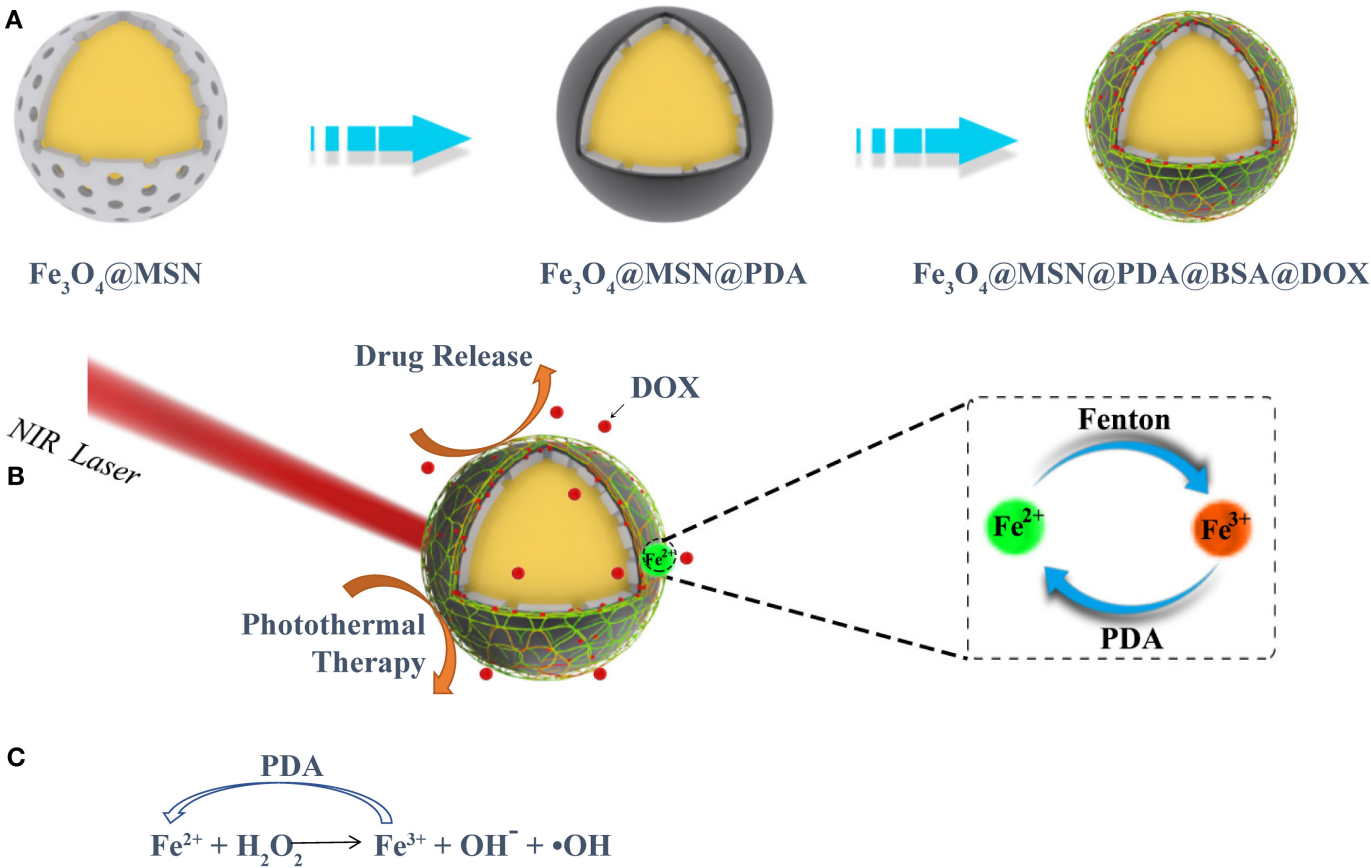
**(A)** The preparation procedures of the FMPBs, **(B)** the ability of FMPBs photothermal treatment and CDT, **(C)** the repeated redox reaction between FMPBs and PDA.

In the tumor microenvironment (TME), special metabolic pathways compared to normal cells cause it to become a place rich in large amounts of hydrogen ions and high reduction (Wu et al., 2019). Unlike normal cells, in the mitochondria of tumor cells, high concentrations of superoxide dismutase cause excessive H_2_O_2_ in the cells (Huo et al., 2017; Dong et al., 2019). Designing nanomaterials with CDT specificity in the TME has become a new way for tumor-targeting therapy. In CDT, nanomaterials precisely catalyze the reaction of H_2_O_2_ with Fe^2+^ (Fenton reaction), which in turn produce cytotoxic hydroxyl radicals (·OH) to kill tumor cells. Magnetic nanoparticles with tumor cell targeting, such as Fe_3_O_4_, are considered to be promising nanocatalytic enzymes that can generate ·OH for CDT (Feng et al., 2018b). In the Fenton reaction, magnetic Fe_3_O_4_ nanoparticles provide the Fe^2+^ needed for the reaction (Feng et al., 2018b). The continuous progress of the Fenton reaction depends on the conversion of Fe^3+^ to Fe^2+^ by materials with nanocatalytic capabilities. Because of its drug delivery ability and magnetocaloric effect, the superparamagnetic Fe_3_O_4_ nanomaterials have received extensive attention (Zhu et al., 2013). Moreover, Fe_3_O_4_ can provide a large number of Fe^2+^ to support the Fenton reaction for CDT of tumors (Zhao et al., 2018). Among many photothermal conversion materials, PDA has received special attention because of its good biocompatibility (Zhu et al., 2013). With the PDA coating, the nanoparticles have excellent PTT capability and good biocompatibility (Dai et al., 2017). Furthermore, the heat generated by PTT can further accelerate the release of the loaded drug in the nanoparticles. Moreover, PDA has a mild reducibility, which provides an inexhaustible motive force for the reduction of Fe^3+^ to Fe^2+^, promotes the production of ·OH in tumor cells, and achieves the goal of killing tumor cells (Schemes 1B,C).

To combine the above functions in a single nanoplatform, we designed a novel nanosystem having multiple therapeutic efficacies. As shown in Scheme 1A, superparamagnetic Fe_3_O_4_ nanospheres with uniform size and good dispersibility were first prepared by the hydrothermal synthesis method (Gao et al., 2013; Jin et al., 2019). On the surface of the prepared Fe_3_O_4_ nanospheres, a thin layer of SiO_2_ was coated (Tang and Cheng, 2013; Huang et al., 2017). Then, a thick layer of mesoporous silica nanospheres (MSNs) was evenly coated on SiO_2_ (Tran et al., 2018). Finally, PDA and bovine serum albumin (BSA) were coated on the outer layer of the material, to form the biocompatible (Hu et al., 2010; Wang et al., 2011) magnetic Fe_3_O_4_@MSN@PDA@BSA nanoparticles (defined as FMPBs).

## Materials and Methods

### Materials

Sodium acetate (anhydrous), 3,3′5,5,5′-tetramethyldiphenylamine (TMB), ferric chloride hexahydrate (FeCl_3_·6H_2_O), dopamine hydrochloride, poly(4-benzene) ethylene sulfonic acid–cobalt maleate sodium salt (PSSMA), sodium dihydrogen phosphate, triethanolamine (TEA), doxorubicin hydrochloride (DOX), Dulbecco modified eagle medium (DMEM), calcium fluoride (CaF_2_), hexadecyl trimethyl ammonium bromide (CTAB), tetraethoxysilane (TEOS), acetone, ethanol, citric acid, disodium hydrogen phosphate, BSA, and sodium fluoride (NaF) were bought from Aladdin Reagents (China). Acetic acid, trisodium citrate, and citric acid were purchased from Sinopharm Chemical Reagent Co., Ltd. (China). Capstone FS-66 reagent was purchased from Sigma–Aldrich (USA). Human colon cancer cells (HT29) were purchased from the Institute of Biochemistry and Cell Biology, Chinese Academy of Sciences (Shanghai, China).

### Preparation of the FMPBs Nanoparticles

For the synthesis of Fe_3_O_4_, 1.50 g of FeCl_3_·6H_2_O and 1.0 g of PSSMA were added to 120 mL of ethylene glycol, heated to 70°C, and fully dissolved for 0.5 h. Then, 4.50 g sodium acetate and 0.75 g of CaF_2_ were added to the mixed solution and continuously stirred for 2 h until fully dissolved. Using the hydrothermal synthesis method, the above-40-mL hot solution was transferred to a polytetrafluoroethylene autoclave with a capacity of 100 mL and reacted at 210°C for 10 h. The Fe_3_O_4_ nanospheres were separated using the magnet and washed three times with ethanol and water (5 min of sonication in each time). Finally, the obtained Fe_3_O_4_ nanospheres were dispersed in water. For the synthesis of Fe_3_O_4_@SiO_2_, Fe_3_O_4_ was dispersed in water (0.1 g/mL) and sonicated for 5 min; 50 mL of the above solution was then mixed with 2.5 mL of 1% NaF solution. Then, a mixture solution of TEOS and ethanol with a mixing ratio of 1:10 was added to the above solution. The resultant solution was stirred quickly for 15 min and blended gently for 8 h using the Votex (MX-F). After the reaction, the solution was sonicated and washed three times with ethanol. For the synthesis of Fe_3_O_4_@MSN, 32 g CTAB and 6 mL TEA were dissolved in 500 mL deionized water. Fifty milliliters of capstone FS-66 (0.2 g/mL) dissolved in acetone was slowly added to the above solution. The mixed solution was stirred at a constant speed for 1 h. 25 mL of TEOS was quickly added to the mixed solution and shaken for 100 s. The obtained mixed solution was added to the Fe_3_O_4_@SiO_2_ solution and slowly stirred for 6 h. The Fe_3_O_4_@MSN nanospheres were separated using the magnet and washed three times with ethanol. To coat PDA, 100 mg of dopamine hydrochloride was dissolved in 50 mL phosphate-buffered saline (PBS) (pH 8.5), which was then added with 100 mg of Fe_3_O_4_@MSN, and sonicated for 5 min and stir slowly for 6 h. To link with BSA, 100 mg BSA was added to the above solution, and then stirring was continued for 6 h. After magnetic separation of the above solution, the product was washed three times with water, lyophilized, and stored at 4°C.

### Characterizations

Scanning electron microscope (SEM, Zeiss Merlin Compact) was used to observe the morphology and size of the prepared nanoparticles. X-ray energy-dispersive spectroscopy (EDS, X-MAX-20mm2) was used to determine the elemental composition of the nanoparticles. A transmission electron microscope (TEM) image was taken using a Talos F200X microscope. Thermal gravimetric analysis (TGA, Shimadzu TGA-50) was used to detect the mass percentage of different substances of FMPBs. Quantum Design PPMS-9 (USA) was used to measure the magnetic property of nanoparticles. The X-ray diffraction (XRD) pattern of FMPB nanoparticles was measured by Bruker/D8ADVANCE (DE). Using TriStar II 3020 (USA), the Bernauer–Emmett–Teller (BET) pore size distribution and diameter of FMPB nanoparticles were tested. The dynamic light scattering (DLS) diameter and zeta potential of FMPBs at 25°C were measured by NanoZSZEN3600 (Malvern Instruments). The photothermal conversion performance of FMPB nanoparticles was tested using a laser-producing setup (Shanghai Connor Fiber Co., Ltd). The Fourier transform infrared (FTIR) spectrum of FMPBs was measured by Nicolet Nexus 670. The FTIR data were collected in the range of 500 to 4,000 cm^−1^. The Hitachi spectrometer was used to monitor the Fenton response of FMPBs.

### Catalytic Activities of FMPBs

Using TMB as a substrate, the effect of different substances on the Fenton reaction was studied. TMB (0.8 mM), H_2_O_2_ (5 mM), Fe_3_O_4_@MSN, Fe_3_O_4_@MSN@PDA, and FMPBs (1 mg/mL) were mixed and reacted for 1 min. The absorbance of different coated nanoparticles at the same concentration was measured to determine whether ·OH was produced. To study the effect of PDA on Fenton reaction, 1 mg/mL of Fe_3_O_4_, Fe_3_O_4_@MSN, Fe_3_O_4_@MSN@PDA, or FMPBs was added to TMB/H_2_O_2_, and the solution absorbance at 651 nm was immediately recorded using the ultraviolet, visible, and NIR (UV-vis-NIR spectrometer, HITACHI U-3900). The measurement lasted for 300 s. To study the effect of H_2_O_2_ concentration on the production of ·OH, different concentrations of H_2_O_2_ (concentrations of 2.5, 5.0, 10, 25, and 50 μg/mL) were reacted with FMPBs, and the absorbance of the mixed solution at 651 nm was measured using the UV-vis-NIR spectrometer. To study the effect of temperature on ·OH, the spectrometer was used to measure the absorbance of the mixed solution at 651 nm at different temperatures. To study the effect of pH on the generation of ·OH, the spectrometer was used to measure the absorbance of the mixed solution at 651 nm at different pHs. The measurement must last for 300 s.

### Photothermal Conversion Performance of FMPBs

The absorption of FMPBs in the NIR range was detected by UV-vis-NIR. The NIR laser (wavelength = 808 nm, 1 W/cm^2^) was used to continuously irradiate the FMPBs solutions with different concentrations (0.1, 0.2, 0.5, and 1 mg/mL, 200 μL) for 500 s. To study the power density–dependent temperature profiles of FMPBs, an NIR laser (808 nm) with different power densities (0.2, 0.5, 0.8, and 1.0 W/cm^2^) was used to continuously irradiate the FMPBs aqueous solution (200 μL of 1 mg/mL) for 500 s. To prove the long-term photothermal durability, the FMPBs were stored at room temperature in water (1 mg/mL) for 15 days and continuously irradiated with NIR laser (wavelength = 808 nm, 0.8 W/cm^2^) for 600 s. Using a laser with a power density of 0.8 W/cm^2^ and continuous irradiation for 15 min, the photothermal conversion efficiency of FMPBs was measured. The selected concentration of FMPBs is 0.5 mg/mL, and the volume is 200 μL. The photothermal conversion efficiency (η) of FMPB is calculated according to the formula (1). The photothermal stability of FMPBs was studied by continuously irradiating for five consecutive cycles and recording temperature changes during temperature rise and fall (power density: 0.8 W/cm^2^). In the above experiments, the FLIR E60 thermal imaging camera was used to record the temperature change value and thermal image of the irradiated solution.

(1) $$\begin{array}{l} \eta =\frac{\text{h}\text{S}\left({\text{T}}_{\text{max}}-{\text{T}}_{\text{s}\text{ur}\text{r}}\right)-{\text{Q}}_{\text{d}\text{i}\text{s}}}{\text{I}\left(1-10^{\text{A}\lambda }\right)} \end{array}$$

### *In vitro* Cytocompatibility Examination and Hemocompatibility

FMPBs and HT29 cells were incubated together to study the biocompatibility. The DMEM was supplemented with 10% fetal bovine serum and 100 units/mL of penicillin and 0.1 mg/mL of streptomycin. Cells cultured in the above solution were placed in a humidified 37°C incubator. The cells prepared above were cultured in 96-well-plates (8,000 cells per well) for 24 h. FMPBs with different concentrations were added to the wells, and the number of treated cells was quantitatively studied using the CCK-8 kit after 48 h of continuous cultivation. The above experiment also requires the use of live/dead kits to stain the cells and use phase-contrast microscopy (Leica DM IL LED) to qualitatively study the morphology of HT29 cells.

Kunming mouse red blood cells (mRBCs) were kindly provided by Xinhua Hospital, Shanghai Jiaotong University School of Medicine. For *in vitro* hemocompatibility assay, 0.4 mL mRBCs were mixed with 1.2 mL FMPBs and incubated at 37°C for 2 h (final FMPB concentration: 0.5, 1.0, 1.5, and 2.0 mg/mL). The mRBCs mixed with water or PBS were set as the positive or negative control. After that, the supernatant of the above solution was collected by centrifugation, and the absorbance at 570 nm was detected using UV-vis-NIR spectrophotometer. The hemolytic percentage (HP%) was calculated according to the calculation formula (2). All animal experiments are in compliance with the policies of the Ministry of Health under the guidance of Changhai Hospital of the Second Military Region various animal experiments.

(2) $$\begin{array}{l} \text{H}\text{P}\left(\%\right)=\frac{\left({\text{D}}_{\text{t}}-{\text{D}}_{\text{n}\text{c}}\right)}{\left({\text{D}}_{\text{p}\text{c}}-{\text{D}}_{\text{n}\text{c}}\right)}\times 100\% \end{array}$$

### Determination of Drug Loading Efficiency

The *in vitro* drug loading study was performed as follows: FMPBs with different concentrations are added to the solution of DOX and stirred for 24 h in the dark. The FMPB nanoparticles were washed twice with PBS and centrifuged at 10,000 rpm for 5 min. The supernatant is unadsorbed DOX, and its absorbance at 480 nm was measured using the UV-vis-NIR spectrophotometer to calculate the concentration of DOX (Zhao et al., 2018; Wu et al., 2019). Formula (3) and formula (4) were used to calculate the loading percentage and loading efficiency of DOX, respectively. *W*_*t*_ is the mass of used DOX. *W*_*s*_ is the mass of the unadsorbed DOX. *W* is the total mass of the carrier and the drug.

(3) $$\begin{array}{l} \text{Loadin}\text{g }\text{p}\text{ercentag}\text{e}\;\left(\text{\%}\right)=\frac{{\text{W}}_{\text{t}}-{\text{W}}_{\text{s}}}{\text{W}}\times 100\text{\%} \end{array}$$

(4) $$\begin{array}{l} \text{Loadin}\text{g }\text{efficienc}\text{y}\;\left(\text{\%}\right)=\frac{{\text{W}}_{\text{t}}-{\text{W}}_{\text{s}}}{{\text{W}}_{\text{t}}}\times 100\text{\%} \end{array}$$

### *In vitro* Drug Release Studies

*In vitro* drug release studies were performed using the dialysis bag method at two different temperatures of 37° and 47°C or different pH of 6.4 and 7.4. DOX-loaded FMPBs of the same concentration were added to PBS (pH 7.4) and citrate buffer (pH 6.4). The same volume (1 mL) of the above solution was transferred into a dialysis bag (MWCO: 14KD, width 44 mm) and placed in a plastic tube, which was then incubated in a different release buffer at 37° or 47°C with shaking. At different time intervals, the released solution (1 mL) was removed, and 1 mL of the corresponding buffer was added to the solution. The absorbance at 480 nm of the released solution was monitored to calculate the *in vitro* release data and determine the kinetics and mechanism of DOX using equation (5).

(5) $$\begin{array}{l} \text{cumulativ}\text{e}\;\text{releas}\text{e}\left(\text{\%}\right)=\frac{{\text{v}}_{\text{e}}\overset{\text{n}}{\underset{0}{\sum }}{\text{c}}_{\text{i}}}{{\text{v}}_{0}{\text{c}}_{\text{a}}}\times 100\text{\%} \end{array}$$

### *In vitro* Tumor Therapy

In this work, 0.2 mol/L of H_2_O_2_ was added in DMEM, and the pH of solutions was adjusted to 6.4 with HCl. After that, 8,000 cells/well of HT29 cells were cultured with 0.1mL DMEM in 96-well-plate for 12 h. Then, FMPBs in fresh DMEM (0, 0.25, 0.5, and 1 mg/mL) were added in 96-well-plates (*n* = 3). The solution was then irradiated with 808-nm laser (1 W/cm^2^) for 5 min. Finally, the treated cells were incubated for 24 h. CCK-8 kit and dead/live kit were used to study the cell viability according to the instructions. Leica DM IL LED inverted phase-contrast microscope was used to capture the stained cells (live cells showed green fluorescence and dead cells with red).

### Statistical Analysis

The one-way analysis of variance statistical analysis method was used to assess the significance of the assay data, ^*^p < 0.05, ^**^p < 0.01, ^***^p < 0.001.

## Results and Discussion

### Synthesis and Characterizations of FMPBs

**Figure 1:**
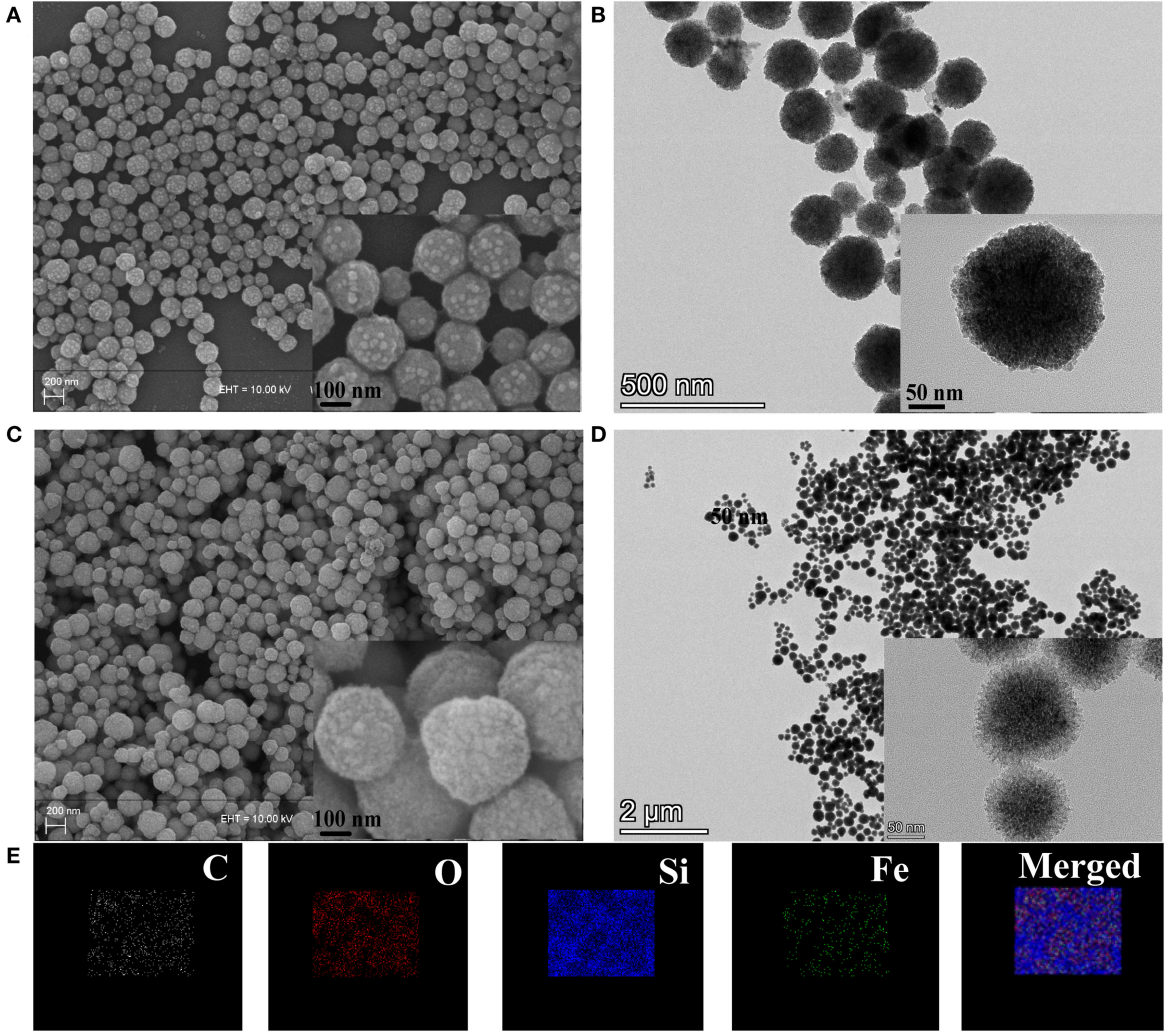
**(A)** SEM images of Fe_3_O_4_, **(B)** TEM images of Fe_3_O_4_, **(C)** SEM images of FMPBs, **(D)** TEM images of FMPBs, inserted images in panels **(A–D)** are the enlarged view, **(E)** elemental distribution mapping of FMPBs.

As shown in Scheme 1A, we prepared nanocarriers with Fe_3_O_4_ as the core and with mesoporous silica, PDA, and BSA as the coating layers. The as-prepared Fe_3_O_4_ nanoparticles were spherical with a diameter of about 150 to 180 nm (Figures 1A,B). Moreover, it was reported that a thin layer of SiO_2_ coated on the Fe_3_O_4_ nanoparticles is necessary for the formation of a mesoporous silica layer (Jin et al., 2019). Thus, before coating mesoporous silica, we coated a thin SiO_2_ shell on the surface of Fe_3_O_4_ in advance. It was found that the FMPB nanospheres have good dispersion and uniform particle size, and the diameter is 250 ± 15 nm (Figure 1C). The TEM images show that the synthesized monodisperse FMPBs exhibit a porous structure, and a layer of PDA and BSA was coated on the surface of the nanospheres (Figures S1, S2). Mesoporous silica was evenly coated on Fe_3_O_4_, and its mesoporous structure is visible. The core-shell structure can be observed more clearly by the high-resolution TEM (Figure 1D). The dispersibility and mesoporous pore structure of nanoparticles are well preserved in FMPBs. Moreover, the hydrodynamic dimensions of Fe_3_O_4_, Fe_3_O_4_@MSN, Fe_3_O_4_@MSN@PDA, and FMPBs were determined as 167 ± 10.4, 203 ± 7.6, 446 ± 50.9, and 380 ± 34.3 nm, respectively (Figure S3). After the PDA coating, the particle size is twice that of the original. This is because the presence of amino groups in the PDA makes it easy to adhere to each other. Notably, the DLS diameter is larger than the real size determined by TEM. This is because the surface of FMPBs is coated with a layer of water molecules in an aqueous solution. There are relevant literature reports that the pore size in the leaky tumor vasculatures is 380–780 nm (Barua and Mitragotri, 2014). Therefore, we have reason to believe that FMPBs with a size of 250 to 380 nm can enter tumor cells. When the outer layer of Fe_3_O_4_@MSN@PDA is grafted with BSA, the amino group is combined with the BSA carboxyl group, and the adhesion is greatly reduced. The zeta potentials of Fe_3_O_4_, Fe_3_O_4_@MSN, Fe_3_O_4_@MSN@PDA, and FMPBs are −58.94 ± 5.64, −87.55 ± 9.11, 6.23 ± 0.172, and −49.92 ± 2.19 mV, respectively (Figure S4). Through the element mapping (Figure 1E) and the EDS analysis (Figures S5, S6), it can be seen that C, O, Si, and Fe in FMPBs are evenly distributed. The above characterizations confirmed that FMPB nanospheres with high dispersibility were successfully prepared.

**Figure 2:**
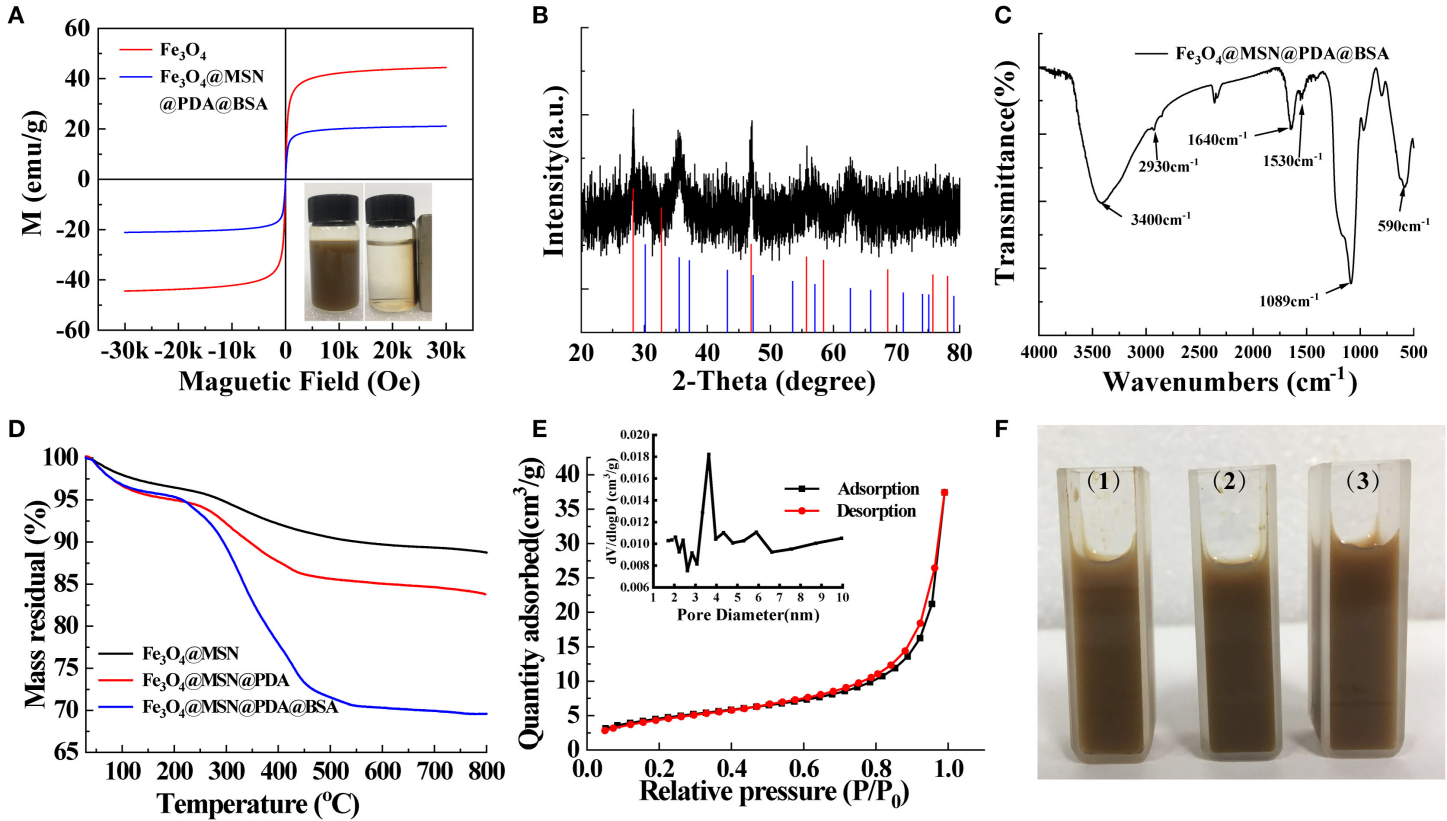
**(A)** VSM of Fe_3_O_4_ and FMPBs, the inserted picture is digital photograph of FMPBs in water that were separated by a magnet; **(B)** powder XRD pattern of FMPBs containing CaF_2_ and Fe_3_O_4_; **(C)** FTIR spectra of FMPBs; **(D)** TGA curves of different nanoparticles; **(E)** BET and BJH measurement results for FMPBs; **(F)** photographic image of FMPBs that were dissolved in water (1), PBS (2), and DMEM (3).

The magnetism, pore size, and coating effect of the material were then analyzed. The magnetic properties of Fe_3_O_4_ and FMPBs were analyzed with a vibrating sample magnetometer (VSM) at room temperature (Figure 2A). It can be seen from the magnetization curve that the saturation magnetizations of Fe_3_O_4_ and FMPBs are 44.43 and 21.1 emu/g, respectively. The saturation magnetization of the FMPBs is smaller than Fe_3_O_4_ NPs. This can be attributed to that the non-magnetic MSN/PDA/BSA layer on the surface of the Fe_3_O_4_ NPs reduced the magnetization. Besides, the S shape of the hysteresis loop indicates that the FMPBs are superparamagnetic. The digital photograph of FMPBs in the water separated by the magnet also confirms its good magnetic properties.

The structure of the as-prepared FMPBs was studied by XRD. As shown in Figure 2B, the diffraction peaks of FMPBs in the figure are consistent with the standard Fe_3_O_4_ (JCPDS No. 19-0629) that was marked by the blue line in the scale. Besides, the peak positions of CaF_2_ (JCPDS No. 99-0051) marked in purple in the diffraction peaks coincided with the formation of CaF_2_ crystals during the preparation of Fe_3_O_4_. The results show that the synthesized magnetic particles contain Fe_3_O_4_ (blue line) and CaF_2_ crystal (red line). Herein, CaF_2_ was used to control the size of Fe_3_O_4_ nanoparticles. Under the coating of PSSMA, the presence of Ca^2+^ makes the Fe_3_O_4_ nanoparticles more uniform and dense during the formation process, and its size has better uniformity and excellent magnetic properties (Jin et al., 2019).

The chemical structure of FMPBs was then determined by FTIR (Figure 2C and Figure S7). The absorption peaks at 590 cm^−1^ can be assigned to Fe-O vibration. The absorption peak at 1,089 cm^−1^ proves the tensile and asymmetric tensile vibration of Si-O-Si, indicating that the structure of MSN is included in FMPBs. The absorption peak at 1,500 cm^−1^ proves that the -C=C- was contained in FMPBs. The broad absorption peak at the range of 3,200–3,600 cm^−1^ is the N-H stretching vibration peak, proving the existence of PDA molecules in FMPBs (Li et al., 2018; Maziukiewicz et al., 2019). The absorption peak of 1,100 cm^−1^ is regarded as the deformation vibration peak of -OH. The absorption peak at 1,530 cm^−1^ can be regarded as the deformation vibration peak of (-NH-) in amide II, whereas the absorption peak at 1,640 cm^−1^ is considered as the vibration peak of –NH_2_) in amide I. The aforementioned several vibration peaks can confirm the existence of BSA molecules in FMPBs. We then used TGA to further monitor the FMPBs surface modification (Figure 2D). According to the TGA curve, the weight loss of FMPBs gradually increased from 40° to 800°C, which was significantly different from Fe_3_O_4_@MSN and Fe_3_O_4_@MSN@PDA. After calculation, the mass percentages of the PDA and BSA layers in FMPBs are 5 and 13%, respectively. Figure 2E shows the adsorption–desorption isotherm curve and pore size distribution of FMPBs measured by BET. The specific surface area and total pore volume of FMPBs were calculated as 16.7108 m^2^/g and 0.057918 cm^3^/g, respectively, and the pore size is about 4.0 nm. The results show that the mesoporous structure of MSN in FMPBs was not significantly changed after being coated with PDA and BSA. FMPBs have good colloidal stability in water, PBS, and DMEM (1 mg/mL, Figure 2F) and exhibit the typical Tyndall effect in the above solutions (Figure S8). Moreover, it is proved that FMPBs can keep stable in water for a long time. The above experiments fully prove that the synthesized FMPBs have good magnetic properties and dispersibility, which provide a reliable guarantee for the long-term tumor treatment.

### Fenton Reaction Induced ·OH Generation

**Figure 3:**
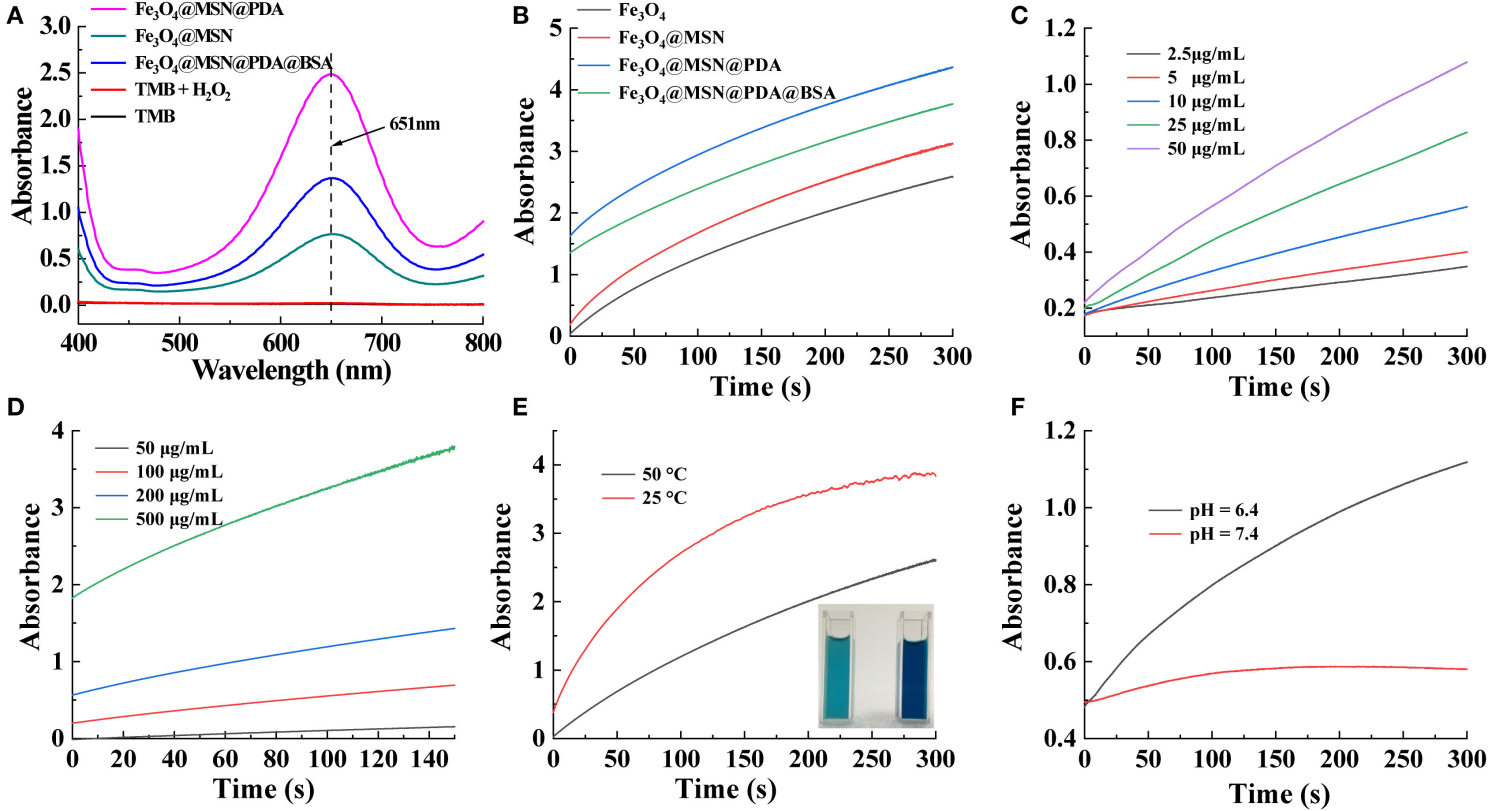
**(A)** UV-vis spectra of the different solution without and with the addition of H_2_O_2_; **(B)** time-dependent absorbance of TMB/H_2_O_2_ solution with Fe_3_O_4_, Fe_3_O_4_@MSN, Fe_3_O_4_@MSN@PDA, and FMPBs, respectively; **(C)** time and H_2_O_2_ concentration-dependent absorbance of FMPBs/TMB solution; **(D)** time-dependent absorbance of FMPBs/TMB/H_2_O_2_ solution with different FMPB concentrations; **(E)** time-dependent absorbance of FMPBs/TMB/H_2_O_2_ solution at the temperature of 25° and 50°C at 651 nm; **(F)** time-dependent absorbance of FMPBs/TMB/H_2_O_2_ solution at different pH at 651 nm.

Different from normal cells, tumor cells are rich in hydrogen ions and H_2_O_2_ (Wu et al., 2019). This specificity in the TME can play an important role in the CDT of tumors. As shown in Figure 3A, because the synthesized Fe_3_O_4_ contains Fe^2+^, the Fenton reaction catalyzed by it can produce a large amount of cytotoxic ·OH. The solution will change from colorless to blue because ·OH can have a significant color reaction with TMB (Wu et al., 2019). The solution after the reaction has a maximum absorption peak at 651 nm, further proving the formation of ·OH in the Fenton reaction (Dong et al., 2019; Wu et al., 2019). However, without of Fe_3_O_4_, no absorption peak was detected of H_2_O_2_ and TMB mixed solution. Owing to the reducibility of the amino group, the PDA could prevent the oxidization of Fe_3_O_4_ and thus support the long-term Fenton reactions (Zeng et al., 2018). Therefore, after adding 1 mg/mL of Fe_3_O_4_@MSN, Fe_3_O_4_@MSN@PDA, and FMPBs to the solution for 1 min, the absorbance of Fe_3_O_4_@MSN@PDA was the largest (Figure 3A). In addition, at the same material concentration, the Fenton reaction rate of the material coated with PDA is greater than that without the PDA coating (Figure 3B). This further proves the role of PDA in promoting the Fenton response. We also examined the influence of H_2_O_2_ on the Fenton reaction (Figure 3C). As the H_2_O_2_ concentration increases, the rate of Fenton reaction also increases. Further, with the increase of FMPB concentration, the Fenton reaction rate is faster (Figure 3D). Besides, the temperature and the acidic environment simulating the TME environment can also promote the production of ·OH. As shown in Figure 3E, the absorbance of FMPBs/TMB/H_2_O_2_ solution at 651 nm at 50°C increases faster than 25°C. The inserted photograph also proves that the higher temperature solution is darker in its color. Similarly, the absorbance value of the solution increases faster in an acidic environment with a pH of 6.4 (Figure 3F). All of these experiments show that the synthesized FMPBs have a good effect in the Fenton reaction and provide good prospects for the TME-specific CDT of tumors.

### Photothermal Conversion Performance

**Figure 4:**
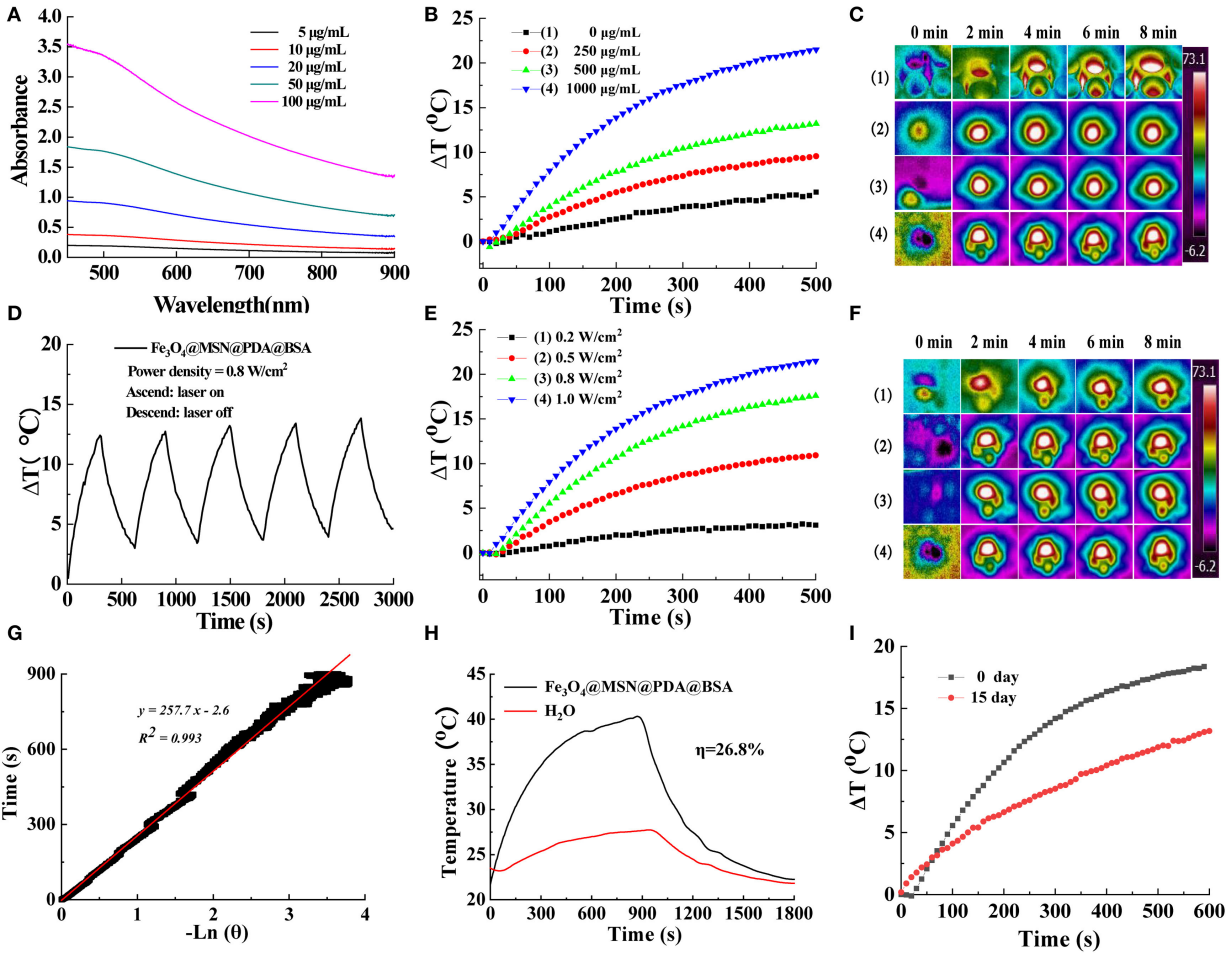
**(A)** UV-Vis-NIR spectra of FMPBs with different concentrations; **(B)** the concentration-dependent temperature curve of FMPBs dispersion during the irradiation of 808-nm laser for 500 s (power density: 1 W/cm^2^); **(C)** corresponding thermal imaging of **(B)**; **(D)** recycling heating profiles of FMPBs; **(E)** power density-dependent temperature curve of 1 mg/mL FMPBs during 500 s of 808-nm laser irradiation; **(F)** corresponding thermal imaging of **(E)**; **(G)** time constant of FMPBs heat transfer under laser irradiation; **(H)** steady-state heating curves of FMPBs and distilled water; **(I)** photothermal conversion profiles of FMPBs dispersed in water after 15 days.

Many studies have shown that PDA with good biocompatibility and adhesion can effectively absorb NIR lasers (Maziukiewicz et al., 2019; Tiwari et al., 2019) and efficiently transform light into heat (Liu et al., 2014; Lin et al., 2018; Chen et al., 2019). Therefore, we studied the light absorption behavior of FMPBs to determine their photothermal conversion ability for PTT of tumors (Maziukiewicz et al., 2019). In the NIR region, FMPBs show absorption of light, and the absorption increases with increasing material concentration (Figure 4A). To study the photothermal properties of FMPBs, we select 808-nm laser to irradiate FMPBs with different concentrations. It was found that the heating rate of the solution increased as the concentration of the FMPBs solution increased. When the concentration of FMPBs is 0.25, 0.5, and 1 mg/mL respectively, the temperature of nanomaterials increases rapidly at 10°, 12.5°, and 22°C under laser irradiation in 500 s (Figure 4B). However, when the material concentration drops to 0, the temperature change is not obvious. We then collected the temperature corresponding photographs of the infrared thermal image, which further provide the evidence of the temperature change of FMPBs (Figure 4C). When the concentration of FMPBs in the solution is 1 mg/mL, the ΔT was 3°, 10°, 17°, and 23°C when the laser intensity was 0.2, 0.5, 0.8, and 1.0 W/cm^2^, respectively (Figure 4E). Figure 4F further provides evidence of temperature changes. We further investigated the photothermal stability of FMPBs. After five NIR laser on/off cycles, there is no significant difference in ΔT, which proves that FMPBs have a good photothermal stability (Figure 4D). Using 808-nm laser with a power density of 0.8 W/cm^2^, the heating transfer time was calculated to be 257.7 s (Figure 4G), and the photothermal conversion efficiency was 26.8% (Figure 4H). Interestingly, the temperature of FMPBs stored at room temperature for 15 days with low-power laser (0.8 W/cm^2^) irradiation still increased by 13°C within 10 min, showing that the material still has good photothermal efficiency after being left for a long time (Figure 4I).

### *In vitro* Cytocompatibility Examination and Hemocompatibility

**Figure 5:**
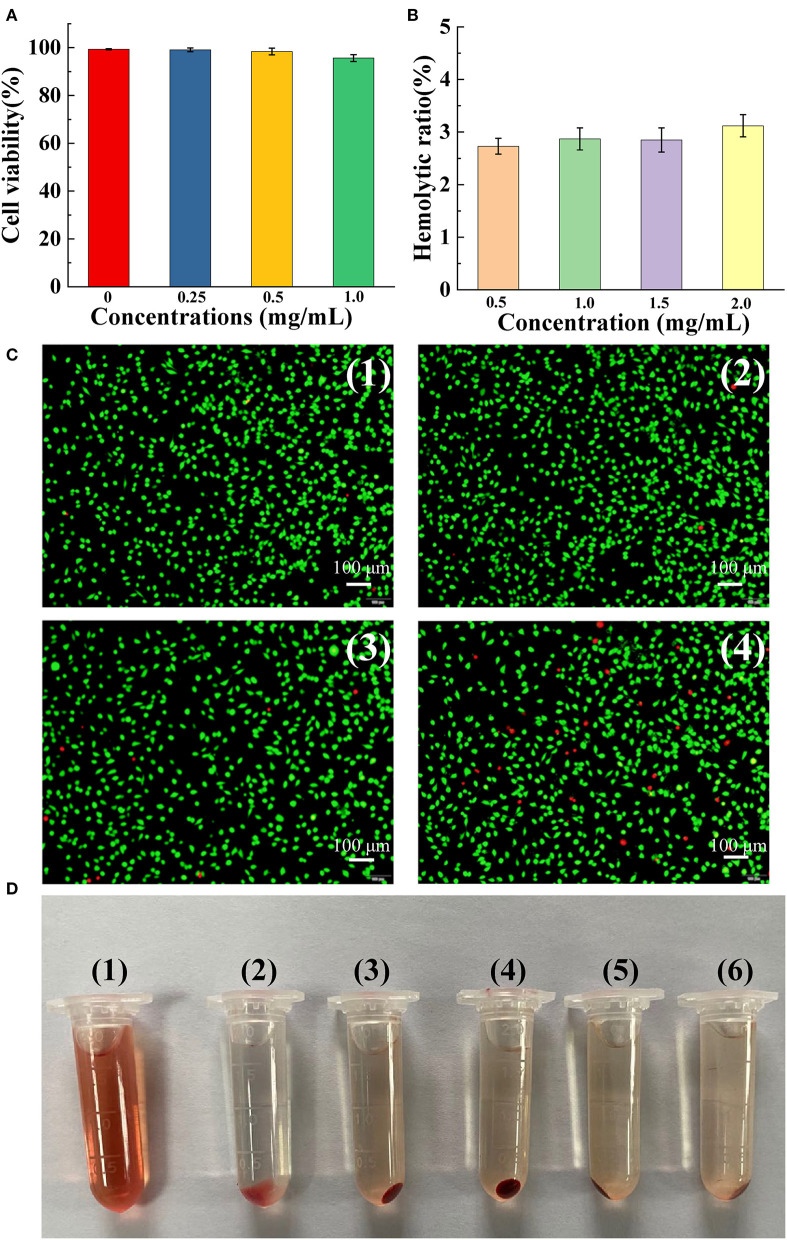
**(A)** Cell viability after incubating HT29 cells with different concentrations of FMPBs for 48 h; **(B)** hemolytic ratio of FMPBs with different concentrations; **(C)** the staining morphology of HT29 cells treated with different concentrations of FMPBs, corresponding to **(A)**; **(D)** digital images of centrifuged mRBCs incubated with different concentrations of FMPBs: (1) water, (2) PBS, (3) 0.5 mg/mL, (4) 1 mg/mL, (5) 1.5 mg/mL, and (6) 2 mg/mL.

We investigated the biocompatibility of FMPBs. As shown in Figure 5A, HT29 was incubated with 0.25 mg/mL FMPBs for 48 h, and its cell viability was 99.12% ± 0.79%. When the concentration of FMPBs increased to 1 mg/mL, the cell viability reached 95.65 ± 1.44%. Figure 5C is the photograph of cell morphology after coincubation corroborating the cell viability assay, which indicates that FMPBs have good biocompatibility. Then, the hemolysis experiment was performed to further evaluate the safety of FMPBs in the clinical application. In this experiment, the hemolysis rate of mRBCs treated with water and PBS was set as 100 and 0%, respectively. As can be seen from the Figure 5B, with different concentrations of FMPBs, the HP is 2.73 ± 0.15%, 2.87 ± 0.21%, 2.85 ± 0.23%, and 3.12 ± 0.21% when FMPB concentrations are 0.5, 1.0, 1.5, and 2.0 mg/mL, respectively. The result of HPs is all less than 5%, indicating that the FMPBs have good blood compatibility. The mRBCs after the centrifugation (Figure 5D) further prove the above conclusion.

### Drug Loading and Release

**Figure 6:**
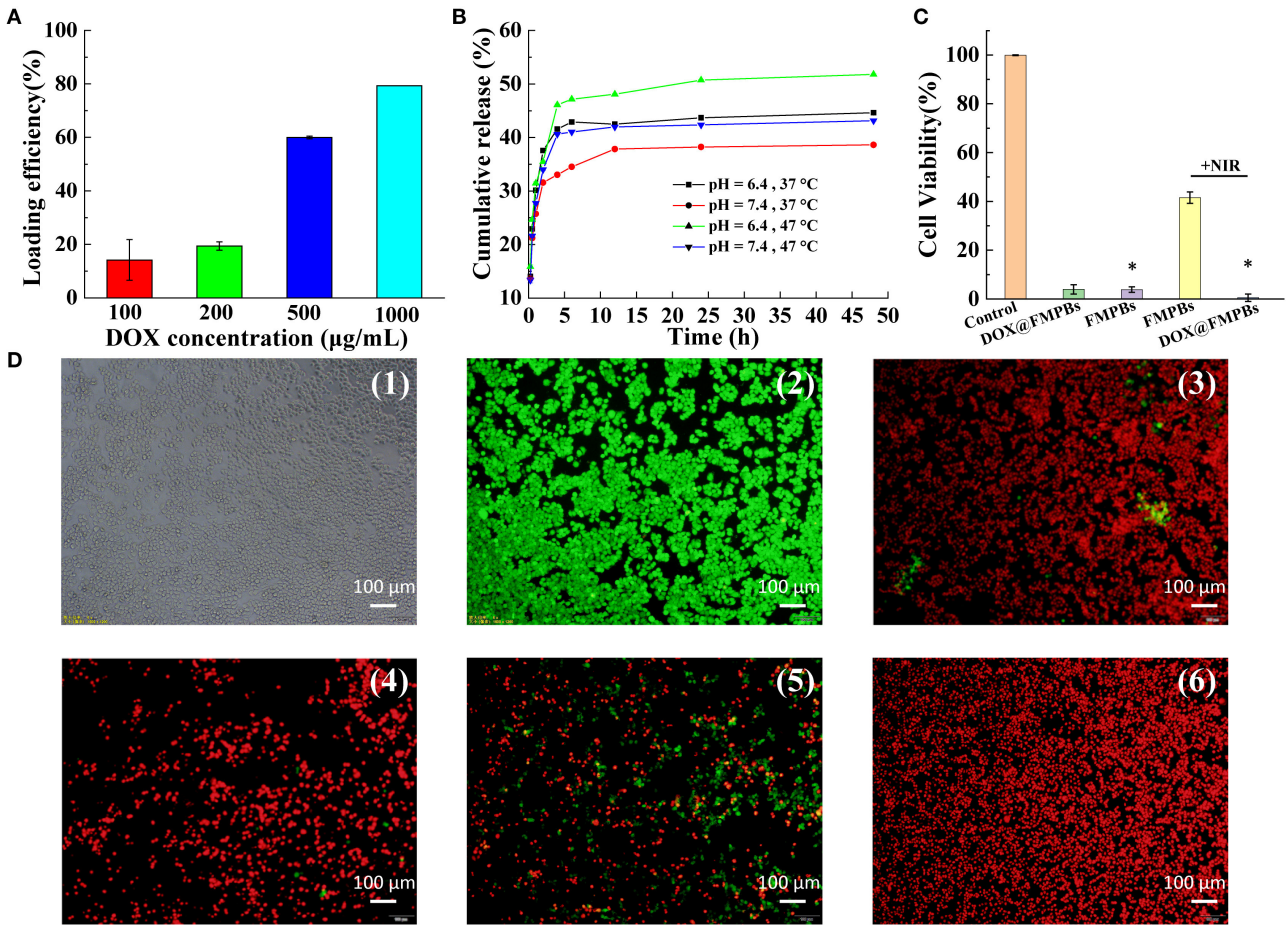
**(A)** Drug loading efficiency of 1 mg/mL FMPBs at different DOX concentrations; **(B)** *in vitro* drug DOX release curve of FMPBs in buffer solutions with different temperature/pH; **(C)** cell viability of HT29 cells after different therapy (* with H_2_O_2_ in DMEM); **(D)** digital images of live or dead HT29 cells corresponding to **(C)**: (1 and 2) control, (3) DOX@FMPBs, (4) FMPBs + H_2_O_2_, (5) FMPBs + NIR, (6) DOX@FMPBs + H_2_O_2_ + NIR.

The porous structure of FMPBs can be used to encapsulate various drug molecules, such as DOX. By dispersing FMPBs and DOX in water, this simple method also easily loads drug molecules into the pores of the material. The drug loading efficiency of nanoparticles was studied. When the drug concentration increased from 0.1 to 1 mg/mL, the loading efficiency of DOX increased from 14.17 ± 7.59% to 79.31 ± 0.003% (Figure 6A). Meanwhile, the drug loading percentage was calculated. As can be seen from Figure S9, as the DOX concentration continuously increased from 100 μg/mL to 1 mg/mL, the drug loading percentage increased from 1.28 ± 0.006% to 39.65 ± 1.56%. According to the results of the above experiments, 1 mg/mL DOX and 1 mg/mL FMPBs were selected to achieve the optimal loading concentration of nanoparticles.

In the study of the pH and temperature-responsive drug release, it was found that the rate of drug release in acidic solutions (pH 6.4) is greater than that in neutral solutions (pH 7.4, Figure 6B). Similarly, the rate of drug release in a solution with a temperature of 47°C is greater than a temperature of 37°C, and the drug release rate increases significantly to 51.8 ± 0.1% at 47°C. In the normal cell environment (pH 7.4, *T* = 37°C), the percentage of DOX released was 36 ± 1.5% (48 h), whereas the percentage of DOX released in the acidic environment was 44 ± 1.4%. The pH response of the FMPB experiment can be considered to the better solubility of the drug in an acidic environment. The temperature response is because the higher temperature increases the thermal motion of the DOX molecule. The DOX release kinetics that relies on the NIR/pH makes FMPBs provide it with a good opportunity to enhance tumor chemotherapy.

### *In vitro* Tumor Therapy

To investigate the *in vitro* tumor therapy of FMPBs, we validated the influence of DOX@FMPBs on the viability of HT29 cells *in vitro* (Figures 6C,D). To mimic the endogenous redox and acid nature of TME, H_2_O_2_ (the final concentration of 10 mM) and HCl (the final pH of 6.4) were added in the DMEM. The concentration of FMPBs was 0.25 mg/mL in all the experiments. As can be seen from Figure 6C, the viability of HT29 cells treated with DOX@FMPBs decreased to 3.95 ± 1.89% after 24 h, indicating that DOX has released from FMPBs *in vitro*. After incubating with FMPBs and H_2_O_2_, the viability of HT29 cells decreased to 3.84 ± 1.14% after 24 h, showing that the FMPBs have reacted with H_2_O_2_ and produced ·OH to kill tumor cells. With laser irradiation (1 W/cm^2^, 5 min), the viability of HT29 cells was 41.53 ± 2.37%, while combined with CDT, PTT, and chemotherapy, all of the HT29 cells had been killed. Figure 6D further supports the cell-killing outcome, where the living cells showing green fluorescence and dead cells are red. These experiments indicate that the FMPBs have an obvious *in vitro* tumor CDT, PTT, and chemotherapy capability.

## Conclusions

In summary, FMPB nanomaterials with high biocompatibility and good magnetic properties were synthesized by a layer-by-layer coating of Fe_3_O_4_. It was found that FMPBs gave a photothermal efficiency of 26.8% and a drug loading efficiency of 79.31 ± 0.003%. PDA was found to play a key role in the entire nanoparticle formation. The high photothermal capability of the nanoparticles not only achieves the heat-induced release by irradiating drug-loaded FMPBs, but also kills tumor cells by increasing the temperature. Meanwhile, the reducibility of the PDA can be utilized to reduce the Fe^3+^ in the Fenton reaction to Fe^2+^. The produced Fe^2+^ can react with H_2_O_2_ in TME and release ·OH to kill tumor cells. Therefore, the structural feature of MSN, the photothermal and the reducibility characteristics of PDA, and the biocompatibility of BSA are integrated into the one nanomaterial, which is anticipated to further promote the development of the precise cancer treatment.

## Data Availability Statement

The raw data supporting the conclusions of this article will be made available by the authors, without undue reservation, to any qualified researcher.

## Author Contributions

CJ designed and completed all tests and wrote this paper. HW completed the biocompatibility and hemocompatibility tests. KL and WH collected the data. MH and SW make revisions to the paper and approve the final paper to be published.

## Conflict of Interest

The authors declare that the research was conducted in the absence of any commercial or financial relationships that could be construed as a potential conflict of interest.

## References

[B1] Barua S. Mitragotri S. (2014). Challenges associated with penetration of nanoparticles across cell and tissue barriers: a review of current status and future prospects. Nano Today 9, 223–243. 10.1016/j.nantod.2014.04.008 25132862 PMC4129396

[B2] Bose R. J. Lee S. H. Park H. (2016). Biofunctionalized nanoparticles: an emerging drug delivery platform for various disease treatments. Drug Discov Today 21, 1303–1312. 10.1016/j.drudis.2016.06.005 27297732

[B3] Bulbake U. Doppalapudi S. Kommineni N. Khan W. (2017). Liposomal formulations in clinical use: an updated review. Pharmaceutics 9:12. 10.3390/pharmaceutics9020012 28346375 PMC5489929

[B4] Chen Y. Zhao J. Wang S. Zhang Z. Zhang J. Wang Y. (2019). Photothermal composite nanomaterials for multimodal tumor therapy under MRI guidance. Chem. Select. 4, 11156–11164. 10.1002/slct.201903481

[B5] Dai Y. Yang D. Yu D. Cao C. Wang Q. Xie S.. (2017). Mussel-inspired polydopamine-coated lanthanide nanoparticles for NIR-II/CT dual imaging and photothermal therapy. ACS Appl. Mater. Interf. 9, 26674–26683. 10.1021/acsami.7b06109 28726368

[B6] Dong Z. Feng L. Chao Y. Hao Y. Chen M. Gong F.. (2019). Amplification of tumor oxidative stresses with liposomal fenton catalyst and glutathione inhibitor for enhanced cancer chemotherapy and radiotherapy. Nano Lett. 19, 805–815. 10.1021/acs.nanolett.8b03905 30592897

[B7] Du B. Ma C. Ding G. Han X. Li D. Wang E.. (2017). Cooperative strategies for enhancing performance of photothermal therapy (PTT) agent: optimizing its photothermal conversion and cell internalization ability. Small 13:1603275. 10.1002/smll.201603275 28112858

[B8] Feng L. Gai S. Dai Y. He F. Sun C. Yang P. (2018a). Controllable generation of free radicals from multifunctional heat-responsive nanoplatform for targeted cancer therapy. Chem. Mater. 30, 526–539. 10.1021/acs.chemmater.7b04841

[B9] Feng L. Xie R. Wang C. Gai S. He F. Yang D.. (2018b). Magnetic targeting, tumor microenvironment-responsive intelligent nanocatalysts for enhanced tumor ablation. ACS Nano 12, 11000–11012. 10.1021/acsnano.8b05042 30339353

[B10] Gao J. Ran X. Shi C. Cheng H. Cheng T. Su Y. (2013). One-step solvothermal synthesis of highly water-soluble, negatively charged superparamagnetic Fe3O4 colloidal nanocrystal clusters. Nanoscale 5, 7026–7033. 10.1039/c3nr00931a 23803791

[B11] Gulzar A. Xu J. Yang D. Xu L. He F. Gai S.. (2018). Nano-graphene oxide-UCNP-Ce6 covalently constructed nanocomposites for NIR-mediated bioimaging and PTT/PDT combinatorial therapy. Dalton Trans 47, 3931–3939. 10.1039/C7DT04141A 29459928

[B12] Hu H. Yu B. Ye Q. Gu Y. Zhou F. (2010). Modification of carbon nanotubes with a nanothin polydopamine layer and polydimethylamino-ethyl methacrylate brushes. Carbon 48, 2347–2353. 10.1016/j.carbon.2010.03.014

[B13] Huang M. Liu L. Wang S. Zhu H. Wu D. Yu Z.. (2017). Dendritic mesoporous silica nanospheres synthesized by a novel dual-templating micelle system for the preparation of functional nanomaterials. Langmuir 33, 519–526. 10.1021/acs.langmuir.6b03282 27989129

[B14] Huo M. Wang L. Chen Y. Shi J. (2017). Tumor-selective catalytic nanomedicine by nanocatalyst delivery. Nat. Commun. 8:357. 10.1038/s41467-017-00424-8 28842577 PMC5572465

[B15] Jahanban-Esfahlan A. Panahi-Azar V. Sajedi S. (2015). Spectroscopic and molecular docking studies on the interaction between N-acetyl cysteine and bovine serum albumin. Biopolymers 103, 638–645. 10.1002/bip.22697 26139573

[B16] Jia H. Z. Zhang W. Zhu J. Y. Yang B. Chen S. Chen G.. (2015). Hyperbranched-hyperbranched polymeric nanoassembly to mediate controllable co-delivery of siRNA and drug for synergistic tumor therapy. J. Control Release 216, 9–17. 10.1016/j.jconrel.2015.08.006 26272764

[B17] Jin Q. Liu L. Zheng Y. Wang S. Huang M. (2019). Synthesis, characterization, and luminescence properties of BiVO4:Eu3+ embedded Fe3O4@mSiO2 nanoparticles. J. Luminesci. 215:116677 10.1016/j.jlumin.2019.116677

[B18] Li M. Sun X. Zhang N. Wang W. Yang Y. Jia H.. (2018). NIR-activated polydopamine-coated carrier-free Nanobomb for *in situ* on-demand drug release. *Adv Sci*. 5;1800155. 10.1002/advs.201800155 30027047 PMC6051140

[B19] Lin L. S. Song J. Song L. Ke K. Liu Y. Zhou Z.. (2018). Simultaneous fenton-like ion delivery and glutathione depletion by MnO2 -based nanoagent to enhance chemodynamic therapy. Angew. Chem. Int. Ed. Engl. 57, 4902–4906. 10.1002/anie.201712027 29488312

[B20] Liu Y. Ai K. Lu L. (2014). Polydopamine and its derivative materials: synthesis and promising applications in energy, environmental, and biomedical fields. Chem. Rev. 114, 5057–5115. 10.1021/cr400407a 24517847

[B21] Maziukiewicz D. Grzeskowiak B. F. Coy E. Jurga S. Mrowczynski R. (2019). NDs@PDA@ICG conjugates for photothermal therapy of glioblastoma multiforme. Biomimetics 4:3. 10.3390/biomimetics4010003 31105189 PMC6477600

[B22] Qin S. Y. Zhang A. Q. Cheng S. X. Rong L. Zhang X. Z. (2017). Drug self-delivery systems for cancer therapy. Biomaterials 112, 234–247. 10.1016/j.biomaterials.2016.10.016 27768976

[B23] Siegel R. L. Miller K. D. Jemal A. (2016). Cancer statistics, 2016. CA Cancer J. Clin. 66, 7–30. 10.3322/caac.21332 26742998

[B24] Song X. Liang C. Feng L. Yang K. Liu Z. (2017). Iodine-131-labeled, transferrin-capped polypyrrole nanoparticles for tumor-targeted synergistic photothermal-radioisotope therapy. Biomater. Sci. 5, 1828–1835. 10.1039/C7BM00409E 28660918

[B25] Tang L. Cheng J. (2013). Nonporous silica nanoparticles for nanomedicine application. Nano Today 8, 290–312. 10.1016/j.nantod.2013.04.007 23997809 PMC3757135

[B26] Tiwari A. P. Bhattarai D. P. Maharjan B. Ko S. W. Kim H. Y. Park C. H.. (2019). Polydopamine-based implantable multifunctional nanocarpet for highly efficient photothermal-chemo therapy. Sci. Rep. 9:2943. 10.1038/s41598-019-39457-y 30814589 PMC6393577

[B27] Tran A. V. Shim K. Vo Thi T. T. Kook J. K. An S. S. A. Lee S. W. (2018). Targeted and controlled drug delivery by multifunctional mesoporous silica nanoparticles with internal fluorescent conjugates and external polydopamine and graphene oxide layers. Acta Biomater. 74, 397–413. 10.1016/j.actbio.2018.05.022 29775731

[B28] Wang S. Li K. Chen Y. Chen H. Ma M. Feng J.. (2015). Biocompatible PEGylated MoS2 nanosheets: controllable bottom-up synthesis and highly efficient photothermal regression of tumor. Biomaterials 39, 206–217. 10.1016/j.biomaterials.2014.11.009 25468372

[B29] Wang S. Yang X. Zhou L. Li J. Chen H. (2020). 2D nanostructures beyond graphene: preparation, biocompatibility and biodegradation behaviors. J. Mater. Chem. B 8, 2974–2989. 10.1039/C9TB02845E 32207478

[B30] Wang S. Zhou L. Zheng Y. Li L. Wu C. Yang H. (2019). Synthesis and biocompatibility of two-dimensional biomaterials. Colloids Surf. Physicochem. Eng. Aspects 583:124004 10.1016/j.colsurfa.2019.124004

[B31] Wang W. Jiang Y. Liao Y. Tian M. Zou H. Zhang L. (2011). Fabrication of silver-coated silica microspheres through mussel-inspired surface functionalization. J. Colloid Interface Sci. 358, 567–574. 10.1016/j.jcis.2011.03.023 21481409

[B32] Wu C. Wang S. Zhao J. Liu Y. Zheng Y. Luo Y. (2019). Biodegradable Fe(III)@WS_2_ -PVP nanocapsules for redox Reaction and TME-Enhanced nanocatalytic, photothermal, and chemotherapy. Adv. Funct. Mater. 29:1901722 10.1002/adfm.201901722

[B33] Xu C. Chen F. Valdovinos H. F. Jiang D. Goel S. Yu B.. (2018). Bacteria-like mesoporous silica-coated gold nanorods for positron emission tomography and photoacoustic imaging-guided chemo-photothermal combined therapy. Biomaterials 165, 56–65. 10.1016/j.biomaterials.2018.02.043 29501970 PMC5880312

[B34] Xu Y. Zhao J. Zhang Z. Zhang J. Huang M. Wang S. (2020). Preparation of electrospray ALG/PDA-PVP nanocomposites and their application in cancer therapy. Soft. Matter. 16, 132–141. 10.1039/C9SM01584A 31774105

[B35] Xue T. Xu C. Wang Y. Wang Y. Tian H. Zhang Y. (2019). Doxorubicin-loaded nanoscale metal-organic framework for tumor-targeting combined chemotherapy and chemodynamic therapy. Biomater Sci. 7, 4615–4623. 10.1039/C9BM01044K 31441464

[B36] Zeng X. Luo M. Liu G. Wang X. Tao W. Lin Y.. (2018). Polydopamine-modified black phosphorous nanocapsule with enhanced stability and photothermal performance for tumor multimodal treatments. Adv Sci. 5:1800510. 10.1002/advs.201800510 30356942 PMC6193171

[B37] Zhao J. Xie P. Ye C. Wu C. Han W. Huang M. (2018). Outside-in synthesis of mesoporous silica/molybdenum disulfide nanoparticles for antitumor application. Chem. Eng. J. 351, 157–168. 10.1016/j.cej.2018.06.101

[B38] Zhou L. Zhao J. Chen Y. Zheng Y. Li J. Zhao J.. (2020). MoS2-ALG-Fe/GOx hydrogel with Fenton catalytic activity for combined cancer photothermal, starvation, and chemodynamic therapy. Colloids Surf. Biointerf. 195, 111243–111243. 10.1016/j.colsurfb.2020.111243 32663712

[B39] Zhu H. Tao J. Wang W. Zhou Y. Li P. Li Z. (2013). Magnetic, fluorescent, and thermo-responsive Fe(3)O(4)/rare earth incorporated poly(St-NIPAM) core-shell colloidal nanoparticles in multimodal optical/magnetic resonance imaging probes. Biomaterials 34, 2296–2306. 10.1016/j.biomaterials.2012.11.056 23274069

