# Supplementary figures and images for: Magnetic Silica Nanosystems With NIR-Responsive and Redox Reaction Capacity for Drug Delivery and Tumor Therapy
Source: Front Chem. 2020 Oct 22;8:567652. doi: 10.3389/fchem.2020.567652 (PMC7643033; doi:10.3389/fchem.2020.567652)

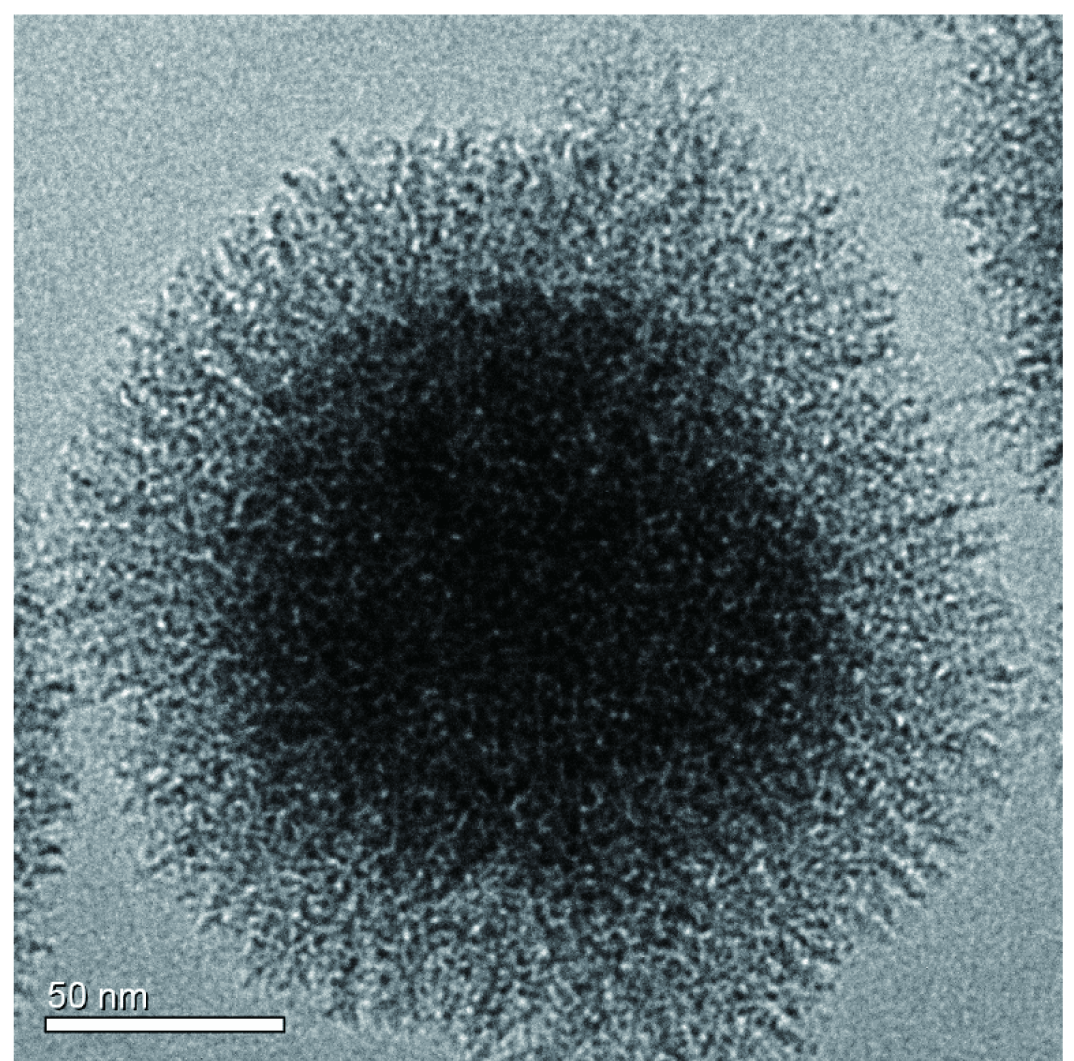

Supplement: Supplementary file 1 [file Image_1.TIF]

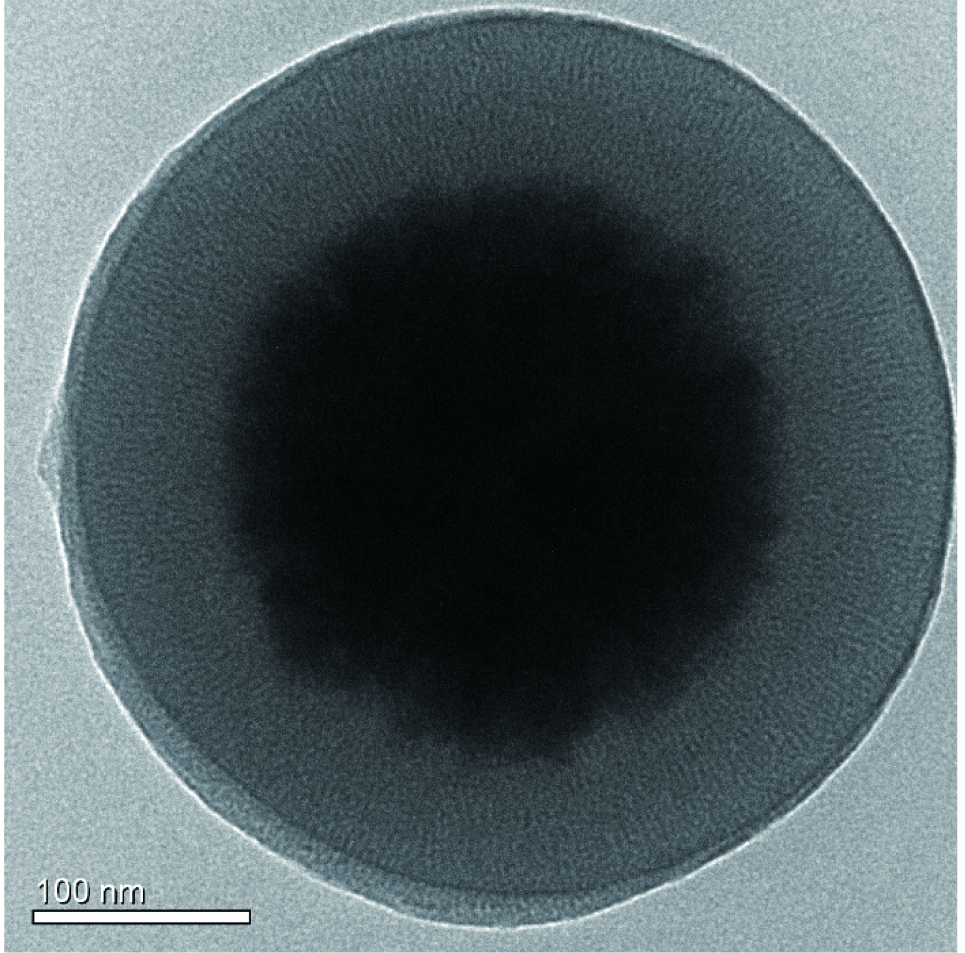

Supplement: Supplementary file 2 [file Image_2.TIF]

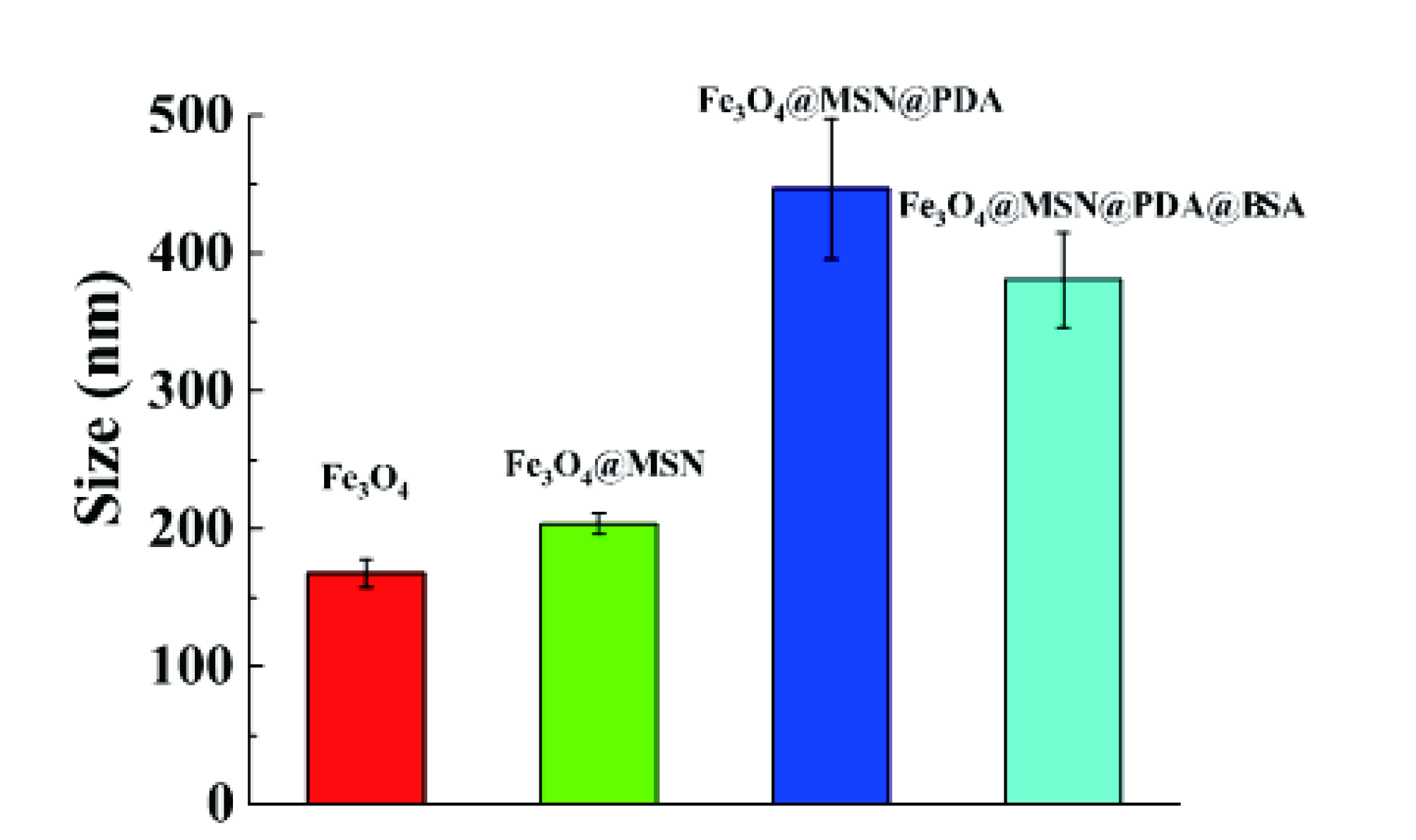

Supplement: Supplementary file 3 [file Image_3.TIF]

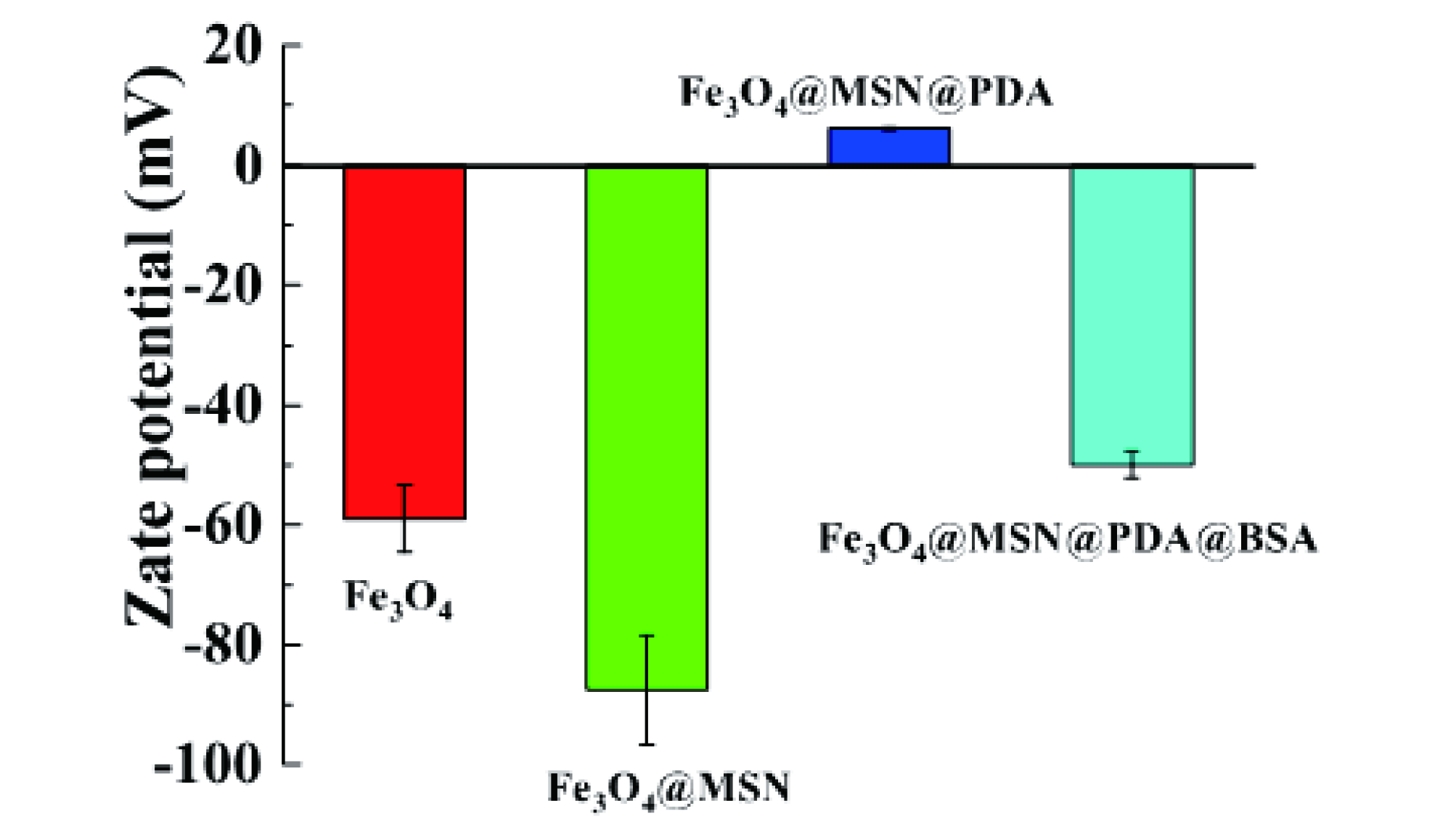

Supplement: Supplementary file 4 [file Image_4.TIF]

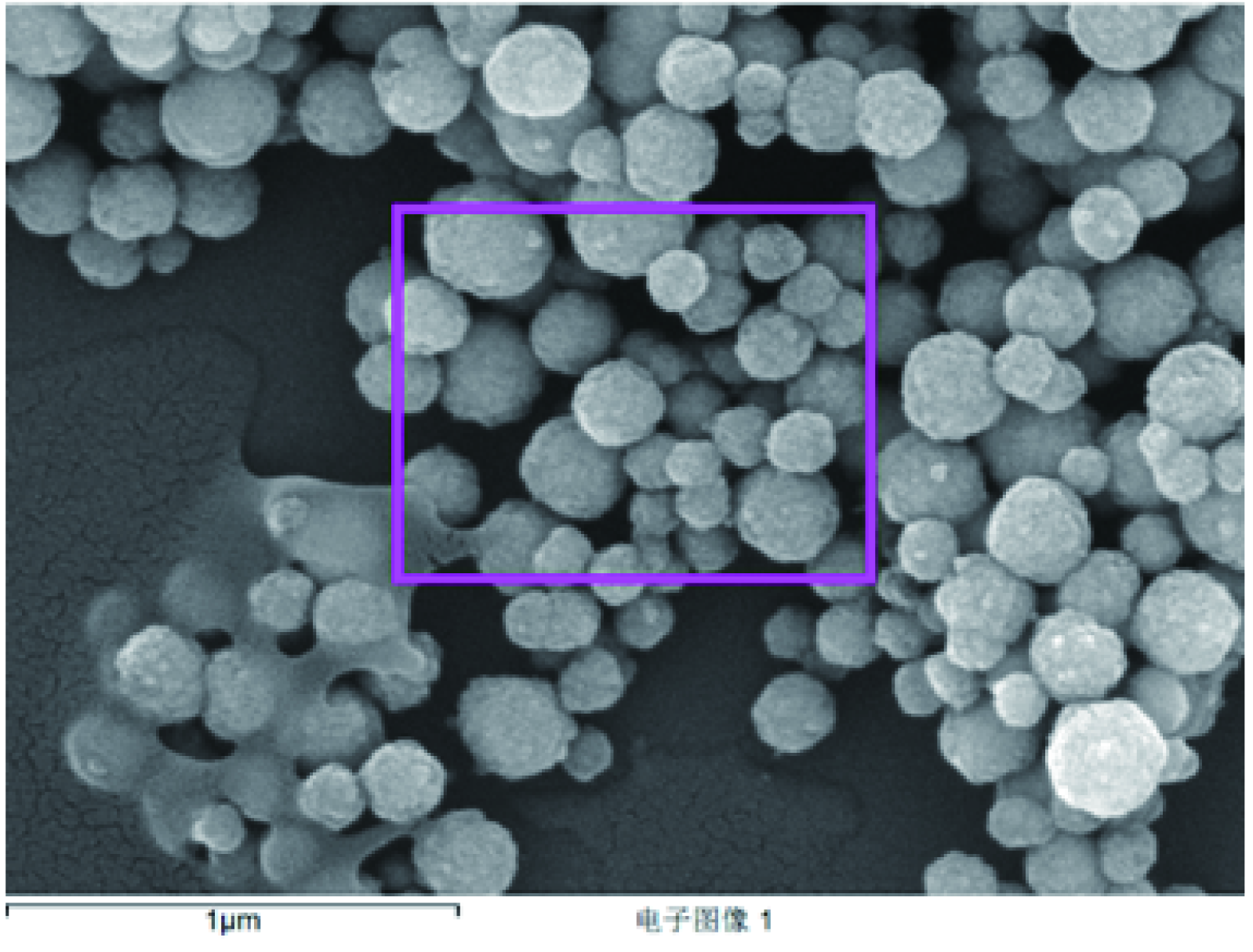

Supplement: Supplementary file 5 [file Image_5.TIF]

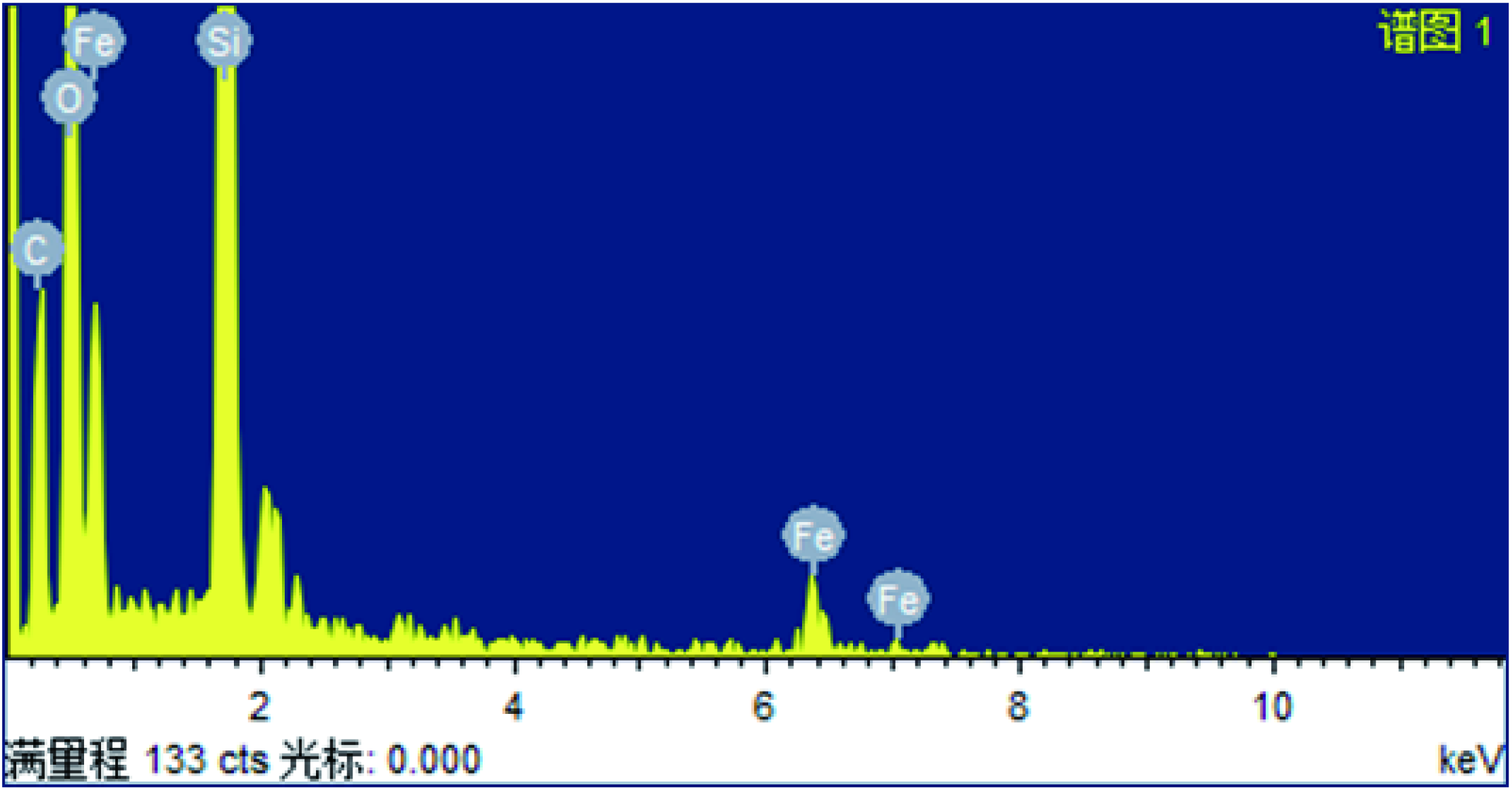

Supplement: Supplementary file 6 [file Image_6.TIF]

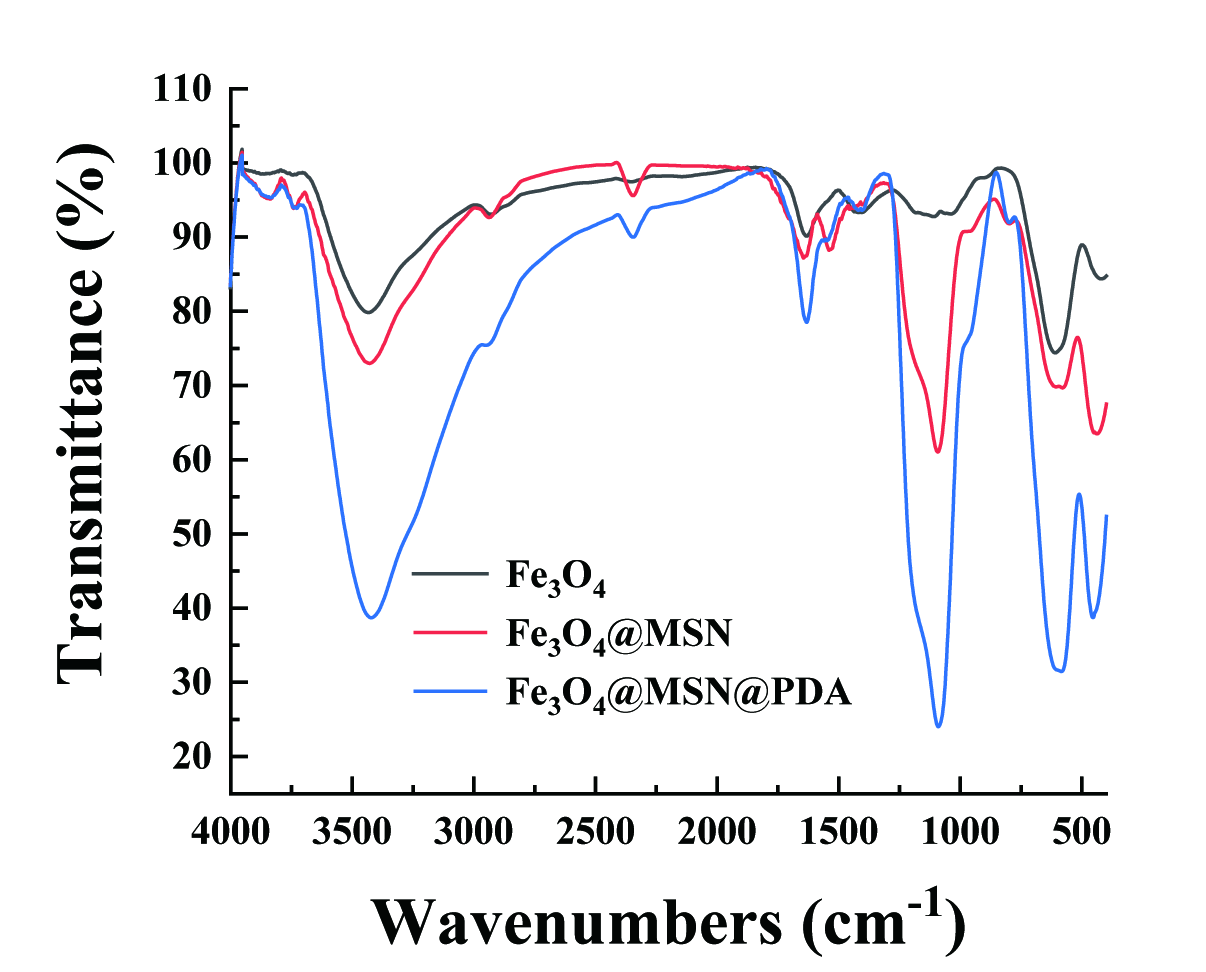

Supplement: Supplementary file 7 [file Image_7.TIF]

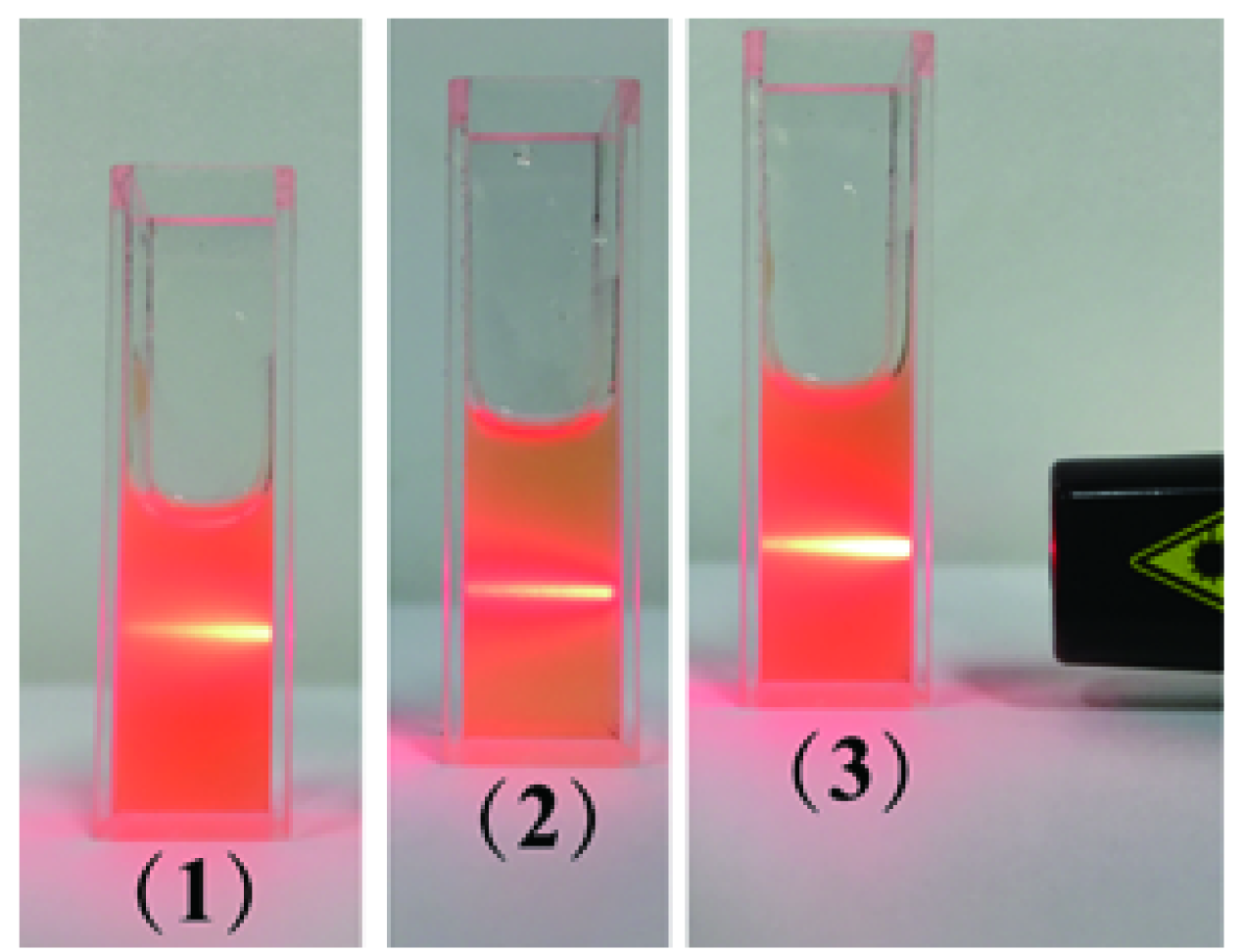

Supplement: Supplementary file 8 [file Image_8.TIF]

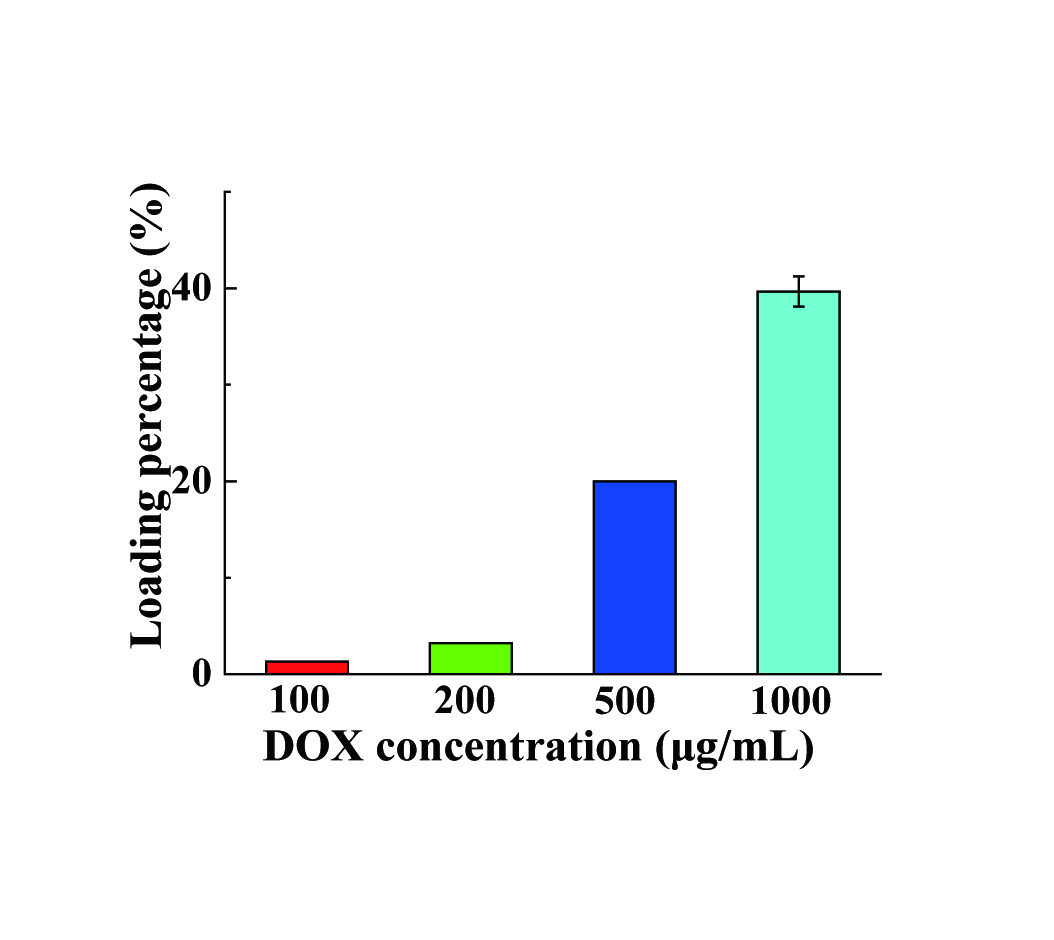

Supplement: Supplementary file 9 [file Image_9.TIF]
