# Supplementary material for: Magnetic Silica Nanosystems With NIR-Responsive and Redox Reaction Capacity for Drug Delivery and Tumor Therapy
Source: Front Chem. 2020 Oct 22;8:567652. doi: 10.3389/fchem.2020.567652 (PMC7643033; doi:10.3389/fchem.2020.567652)
Supplement: Supplementary file 10 [file Data_Sheet_1.docx]

# Supplementary Material





**Figure S1.** TEM of Fe_3_O_4_@MSN





**Figure S2.** TEM of Fe_3_O_4_@MSN@PDA

**Figure S3.** Particle sizes of Fe_3_O_4_, Fe_3_O_4_@MSN, Fe_3_O_4_@MSN@PDA, and FMPBs, respectively;

**Figure S4.** Zeta potentials of Fe_3_O_4_, Fe_3_O_4_@MSN, Fe_3_O_4_@MSN@PDA, and FMPBs, respectively;


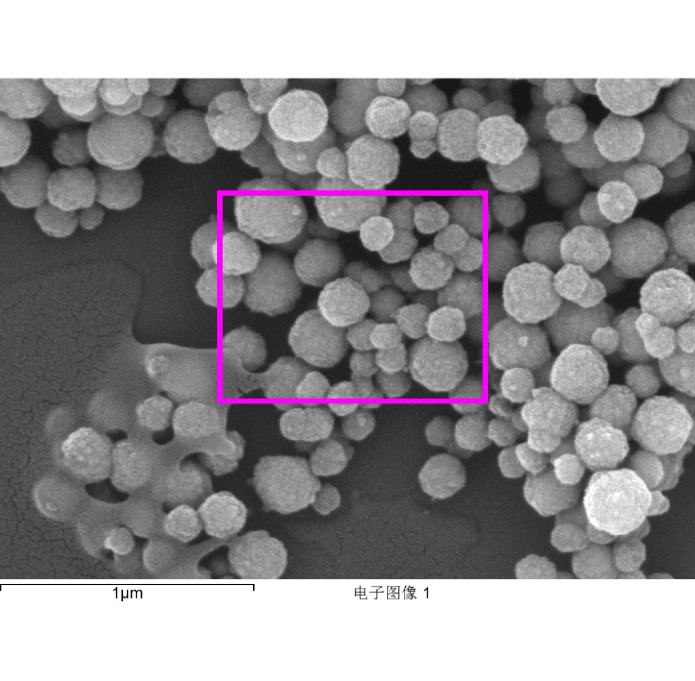


**Figure S5.** SEM of FMPBs


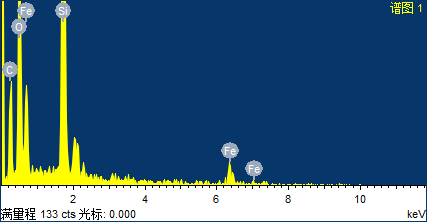


**Figure S6.** EDS spectrum of FMPBs


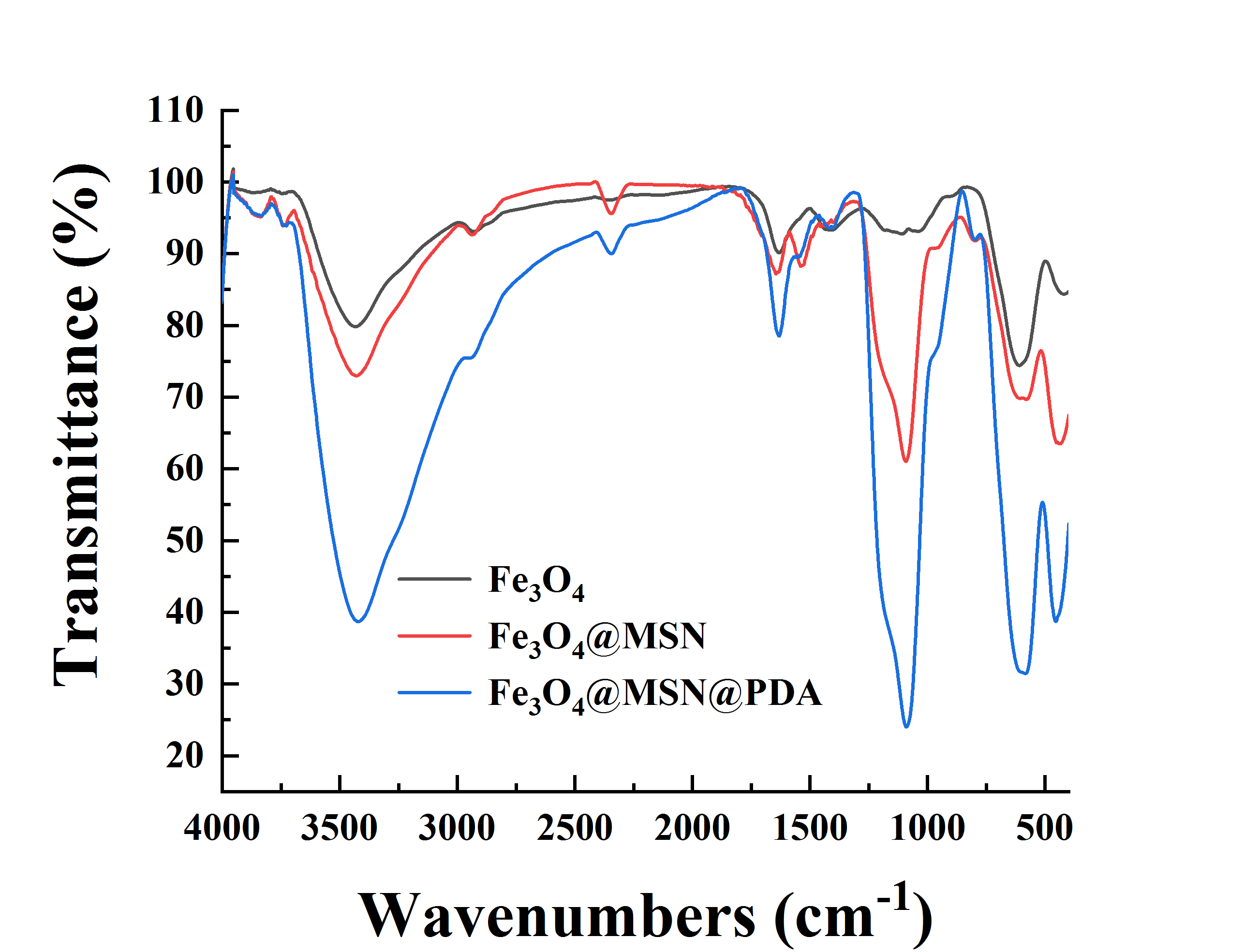


**Figure S7.** FTIR spectra of Fe_3_O_4_, Fe_3_O_4_@MSN, and Fe_3_O_4_@MSN@PDA


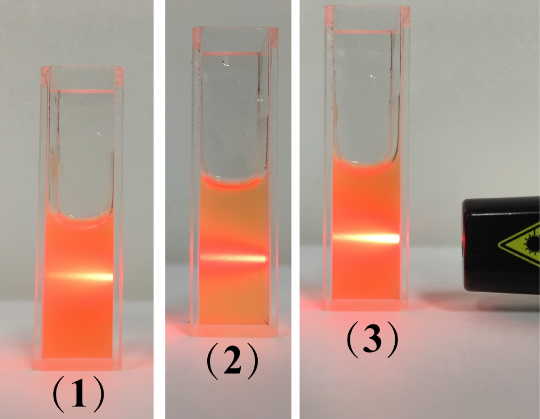


**Figure S8.** Tyndall effect images of (1) Fe_3_O_4_@MSN, (2) Fe_3_O_4_@MSN@PDA, and (3) FMPBs nanocomposites dispersed in water for 24 h.

**Figure S9 .**DOX loading percentage (%)
